# Cordycepin Alleviates Acute Lung Injury by Targeting TAK1 to Inhibit MAPK and NF-κB Signaling Pathways

**DOI:** 10.3390/biom16091279

**Published:** 2026-09-03

**Authors:** Junyan Wang, Kang Zhang, Jingyan Zhang, Zhiting Guo, Guowei Xu, Xiaowei Feng, Xiaoliang Chen, Shuqi Liu, Zhengzhong Luo, Lei Wang, Jianxi Li

**Affiliations:** 1 Traditional Chinese Veterinary Technology Innovation Center of Gansu Province, Key Laboratory of Veterinary Pharmaceutical Discovery, Ministry of Agriculture and Rural Affairs, Lanzhou Institute of Husbandry and Pharmaceutical Sciences, Chinese Academy of Agricultural Sciences, Lanzhou 730000, China; 2 College of Veterinary Medicine, Gansu Agricultural University, Lanzhou 730000, China; 3 College of Veterinary Medicine, Hebei Agricultural University, Baoding 071000, China

**Keywords:** acute lung injury, cordycepin, TAK1, MAPK and NF-κB signaling pathways, LPS

## Abstract

Acute lung injury (ALI) is a common clinical acute respiratory disorder driven primarily by a diffuse pulmonary inflammatory response. Cordycepin (COR) is a bioactive metabolite extracted from the fungus *Cordyceps militaris*, which possesses antioxidant and anti-inflammatory properties. However, its underlying molecular mechanism remains to be elucidated. This study evaluated the therapeutic effect of COR on ALI and the underlying molecular mechanisms. MH-S cells were primed with lipopolysaccharide (LPS) at 1 µg/mL for 24 h and then treated with varying doses of COR for an additional 24 h. WB and RT-qPCR analyses showed that COR inhibited the phosphorylation of TGF-β-activated kinase 1 (TAK1) as well as the key kinases in the MAPK and NF-κB pathways in LPS-induced MH-S cells, as evidenced by decreased TAK1, p38, JNK, IκB-α and P65 expression levels, as well as decreased TNF-α, IL-6, IL-1β, MAP3K7, MAPK8 and MAPK14 relative expression. Six-week-old BALB/c mice were intranasally instilled with LPS at 3 mg/kg, followed 24 h later by oral gavage administration of various concentrations of COR. The results showed that COR can effectively suppress the progression of pulmonary tissue injury; similarly, the expression levels of key proteins and inflammatory factors in the TAK1-MAPK and NF-κB signaling pathways were downregulated. In summary, COR exerts a therapeutic effect on ALI by directly targeting TAK1 to inhibit the activation of the MAPK and NF-κB signaling pathways and concurrently suppressing key inflammatory cytokines.

## 1. Introduction

ALI represents a common acute pulmonary inflammatory disorder characterized by unrestrained local and systemic inflammatory responses. This symptom can be triggered by both direct pulmonary inducers and indirect systemic factors [1]. However, an indispensable structural constituent within the cell wall of Gram-negative bacteria, LPS is widely recognized as a leading etiological factor in the pathogenesis of ALI. Usually, the interaction of LPS with the host receptor triggers multiple signaling pathways and results in the liberation of pro-inflammatory factors, which in turn drive leukocyte homing to the lung and provoke an inflammatory reaction [2,3]. Central to ALI pathophysiology is an intricate cascade featuring disruption of the alveolar capillary membrane, excessive infiltration of neutrophils, the aberrant overproduction of both pro-inflammatory cytokines and chemokines, a process that critically coincides with the hyperactivation of NF-κB, NLRP3 inflammasome, and MAPK [4,5,6]. MAPKs constitute a family of extracellular signal-regulated kinases, including MAPK1, MAPK8 and MAPK14, all of which act as pro-inflammatory mediators [7]. TAK1 is encoded by the gene MAP3K7 and governs multiple pro-survival transduction routes, and its sustained hyperactivity represents a shared molecular hallmark across a spectrum of cancers and immune-mediated inflammatory conditions [8]. TAK1 is recognized as a predominant regulatory kinase that modulates cellular inflammatory networks, with its activity intimately linked to both MAPK and NF-κB signaling modalities [9,10]. Its activation results in the phosphorylation of p38, JNK, and ERK, driving a downstream transduction cascade [11]. Collectively, a mounting body of evidence has implicated its role in the inflammatory pathogenesis of the liver, kidney, and lung [12].

Alveolar macrophages are specialized immune cells residing in the alveolar cavity and constitute the vanguard of the body’s innate immune response. They are responsible for identifying and clearing pathogens and harmful particles, and initiating, regulating, or attenuating local inflammatory responses by secreting cytokines. Alveolar macrophages function as the primary innate immune sentinels in the respiratory bronchioles and airspaces [13]. They play crucial roles throughout the inflammatory process, specializing in clearing cellular debris and resolving inflammation [14]. Under physiological conditions, alveolar macrophages maintain pulmonary immune homeostasis through the clearance of apoptotic cells, the engulfment of inhaled pathogens, and the secretion of moderate amounts of anti-inflammatory cytokines [15]. However, upon exposure to pathogenic triggers such as LPS or viral particles, alveolar macrophages undergo a rapid phenotypic switch from quiescence to a classically activated state with potent pro-inflammatory activity. This state features abundant production of TNF-α, IL-1β, and IL-6, which recruit neutrophils and monocytes into the lung parenchyma, thereby driving the cytokine storm that underlies ALI pathogenesis [16].

Although significant advances have been made in understanding the pathophysiology of ALI, effective preventive and therapeutic strategies remain limited. Nowadays, therapeutic modalities for ALI comprise lung-protective ventilation, fluid balance optimization, mesenchymal stem cell-based strategies, and pharmacotherapy [17]. Regarding pharmacological interventions for ALI, numerous agents have been investigated over the years, including corticosteroids, inhaled vasodilators, surfactant replacement therapy, and various anti-inflammatory agents [18]. However, these anti-inflammatory agents can develop resistance and potential side effects. Consequently, an urgent imperative exists to meet the unmet clinical need for the development of novel therapeutic agents that are both safe and effective in targeting the multiple pathological processes underlying ALI.

**Figure 1:**
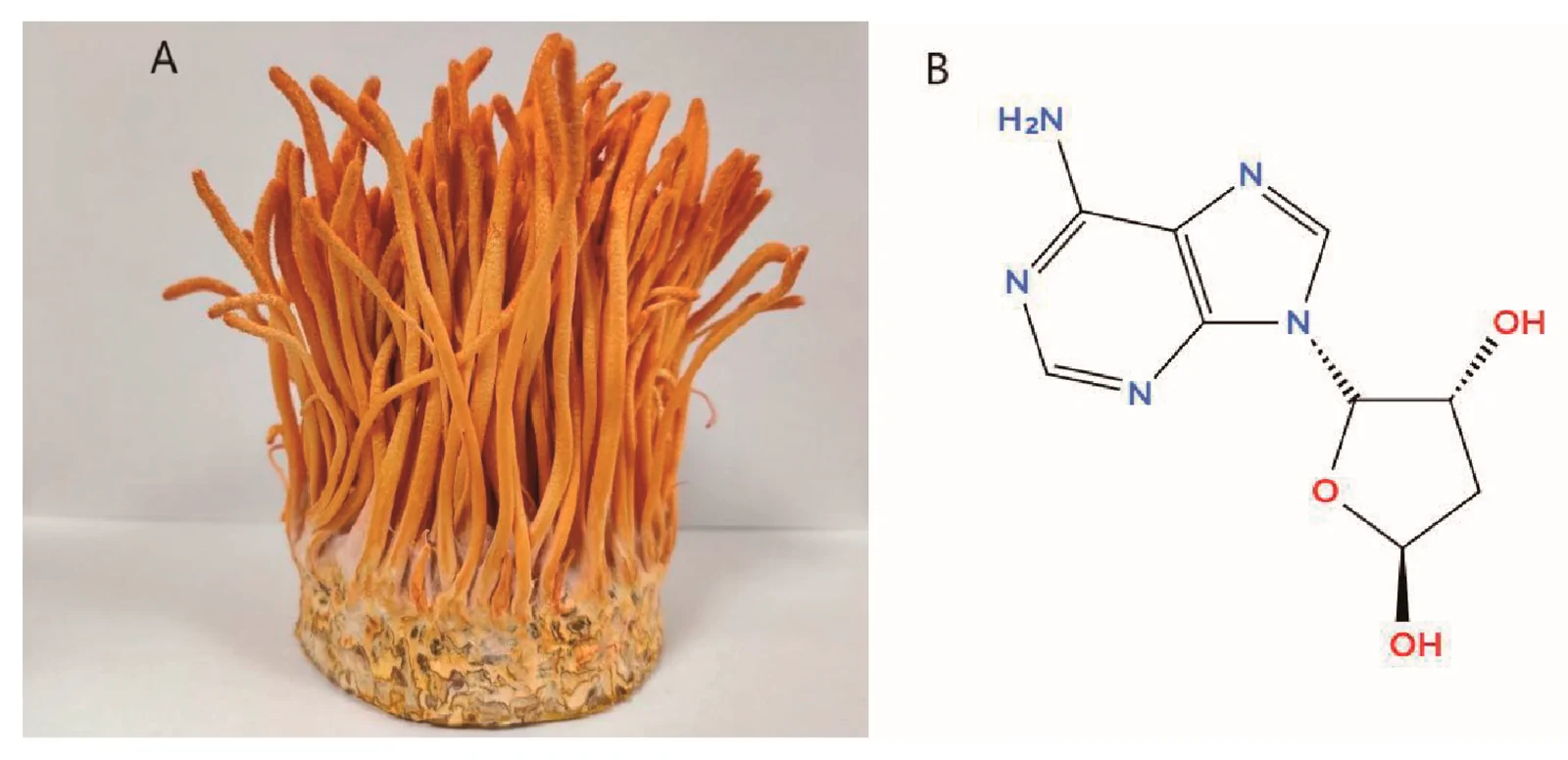
(**A**) Cordyceps militaris. (**B**) Chemical structure of COR.

In recent years, naturally occurring compounds sourced from traditional Chinese medicine (TCM) have attracted escalating research interest as potential ALI interventions, a trend largely attributable to multi-component, multi-target, and multi-pathway pharmacological actions [19,20]. The multi-target pharmacological characteristics of TCM-derived compounds align well with the multifactorial pathogenesis of ALI. TCM active compounds have the capacity to modulate multiple signaling cascades concurrently, thereby achieving synergistic therapeutic effects and potentially reducing the risk of drug resistance [21]. COR is a natural adenosine analog that is one of the bioactive constituents in *Cordyceps militaris* (L.ex Fr.) with the medicinal function in TCM [22] (Figure 1). It harbors a plethora of biological effects, comprising anticancer, anti-inflammatory, antioxidant, and antimicrobial actions [23]. Increasing studies have indicated that COR exerts potential anti-inflammatory effects across various cell populations [24,25]. However, the underlying molecular mechanisms by which COR alleviates ALI have not been definitively identified, and the precise targets lack established causal links to its in vitro and in vivo activity, prompting us to undertake a systematic investigation of its direct binding targets. Therefore, we implement a systematic framework integrating network pharmacology, enrichment analysis, and molecular docking. Leveraging this framework alongside experimental validation, we systematically dissect the molecular underpinnings of COR’s anti-inflammatory action from target and pathway perspectives, and identify candidate key targets as well as key regulatory mechanisms. Its underlying molecular mechanisms hold significant potential to elucidate the molecular mechanism underlying the therapeutic action of COR in ALI and provide a promising candidate for clinical intervention in inflammatory respiratory diseases.

## 2. Materials and Methods

### 2.1. Reagents

COR was sourced from Jiangsu Kangneng Biological Engineering Co. Ltd. (Yangzhou, China). The purity of COR was 98% according to high-performance liquid chromatography analysis. Mouse alveolar macrophage line MH-S cells were purchased from Wuhan Punosai Biotechnology Co., Ltd. (Wuhan, China). LPS (*E. coli*, O111:B4) was purchased from Sigma-Aldrich Trading Co., Ltd. (St. Louis, MO, USA; Shanghai, China).

### 2.2. Cell Culture

The complete growth medium was prepared using RPMI 1640 base medium, 10% fetal bovine serum (FBS), 100 U/mL penicillin and 100 µg/mL streptomycin (Gibco, Carlsbad, CA, USA), and was subsequently stored at 4 °C for future use. The MH-S cells were cultured at a concentration of 5 × 10^5^ cells/mL in complete growth medium and maintained at 37 °C with a 5% CO_2_ atmosphere, and cells were passaged upon density reaching more than 80%.

MH-S cells were systematically randomized into five groups, including the control group (CG), LPS group (MG), MG + 10 µM COR, MG + 1 µM COR and MG + 0.1 µM COR. When the MH-S cells reached 80% confluence, they were first stimulated with the optimal concentration of LPS for 24 h. Subsequently, the cells were treated with varying doses of COR for an additional 24 h. Then, the collected cells and culture supernatant were subjected to WB, RT-qPCR and ELISA analysis, respectively.

To knock down TAK1 expression, cells were transfected with si-NC or si-TAK1, with the siRNAs being supplied by Gene Pharma (Shanghai, China). For overexpression, cells received either the control pcDNA3.1 vector or the TAK1-overexpressing plasmid from Tongyong Biotechnology (Shanghai, China). Cells were plated in media containing 10% FBS to give 30–50% confluency, and transfected using Lipofectamine 2000 (Thermo Fisher Scientific, Carlsbad, CA, USA) as recommended by the manufacturer. After 6 h, the medium was changed, and the cells were stimulated with LPS for 24 h, followed by treatment with COR for an additional 24 h. The cells were then harvested for Western blot and RT-qPCR.

### 2.3. Network Pharmacology Analysis

All the target protein genes of COR were identified using matching the Herb (http://herb.ac.cn, accessed on 26 November 2025), Pub chem (https://pubchem.ncbi.nlm.nih.gov, accessed on 26 November 2025) and Swiss Target Prediction (https://www.swisstargetprediction.ch, accessed on 26 November 2025) system database. The targets of COR against inflammation were screened, and their names were standardized by the UniProt database (https://sparql.uniprot.org, accessed on 26 November 2025) [26]. Disease-associated genes were identified using the GeneCards database (https://www.genecards.org, accessed on 26 November 2025). Overlapping targets, identified via Venn diagram analysis, were considered potential therapeutic targets of COR against inflammation [27]. These targets were uploaded to the STRING platform (https://cn.string-db.org, accessed on 26 November 2025) for the construction of a PPI network, and the resultant interactions were displayed using Cytoscape (v 3.9.0). Lastly, functional enrichment analysis for Gene Ontology (GO) and Kyoto Encyclopedia of Genes and Genomes (KEGG) pathways was carried out using Metascape (https://metascape.org, accessed on 26 November 2025). The top 15 signaling pathways were selected according to their *p*-values and subsequently visualized as bubble diagrams.

### 2.4. Molecular Docking Analysis

The 2D structure of COR was acquired from the PubChem database and then transformed into its 3D form by Chem3D (v 19.1). Protein structures of candidate targets were downloaded from the Protein Data Bank and preprocessed via PyMOL (v 2.5.4) to eliminate ligands and water molecules. Molecular docking was carried out with AutoDock Vina (v 1.5.6), and the interaction patterns were depicted using PyMOL [28,29].

### 2.5. Intervention of COR on LPS-Induced Inflammation Model in Mice

The sample size for each animal group was determined with reference to previous studies in similar LPS-induced ALI and septic models using the TAK1/NF-κB pathway, where *n* = 10/group provided adequate power for detecting significant differences in survival and inflammatory endpoints [30,31]. In addition, we factored in preliminary observations from our laboratory and ethical considerations to reduce animal use. Although a formal a priori power analysis was not performed, the selected group size was considered sufficient to detect biologically meaningful differences based on prior experience and published precedent.

A total of 50 male BALB/c mice (6–8 weeks old, 18–22 g body weight) were obtained from the Lanzhou Veterinary Research Institute, Chinese Academy of Agricultural Sciences (Lanzhou, China). All mice were housed under uniform environmental conditions with fixed cage positions; treatments were administered at a fixed daily time in group-number order, though no randomisation of treatment order or cage rotation was applied. The mice were acclimated for one week prior to the study. Mice were randomly assigned to five groups: CG, MG, high dose group (HDG, MG + 80 mg/kg COR), middle dose group (MDG, MG + 40 mg/kg COR), low dose group (LDG, MG + 20 mg/kg COR). The experimental unit was the individual mouse. Except for CG, other groups were instilled with LPS 3 mg/kg through the nose [32]. After 24 h of stimulation, except for the CG and MG groups, other groups were supplemented with various concentrations of COR solutions via oral gavage, respectively [33]. After a 3-day treatment regimen with COR, the animals were deeply anesthetized using ether. Blood samples were taken only after the loss of corneal and toe-pinch reflexes was assured. The animals were then euthanized, and the lungs were removed for further evaluation.

### 2.6. Histological Analysis

Pulmonary tissues were dissected from experimental mice and immediately immersed in 4% paraformaldehyde (Wuhan China) for 48 h of fixed preservation at 4 °C. After fixation, residual fixative in tissue samples was fully removed via repeated PBS rinsing. Gradient dehydration treatment was performed on the fixed tissues to eliminate internal moisture, and the processed specimens were subsequently embedded in paraffin blocks for long-term preservation and histological detection. Continuous thin sections were prepared from paraffin-embedded lung samples for subsequent staining operations. Paraffin sections of the lung were stained with Hematoxylin–Eosin (H&E) (Solarbio, Beijing, China). Ultimately, microscopic observation was conducted to identify histopathological morphological alterations in lung tissues, and typical visual fields were photographed and systematically stored for subsequent analytical evaluation.

Histological severity of acute lung injury was quantified according to the criteria established [34]. The scoring system evaluated five distinct features: (A) alveolar and (B) interstitial neutrophil accumulation; (C) hyaline membrane formation; (D) proteinaceous exudates within alveolar spaces; and (E) interstitial thickening, each graded from 0, 1, or 2 based on the severity of lung injury. These individual scores were then combined into a weighted composite index using the formula: [(20 × A) + (14 × B) + (7 × C) + (7 × D) + (2 × E)]/(*n* × 100), where *n* represents the number of microscopic fields examined [35]. The resultant ALI score ranges from 0 (no detectable injury) to 1 (severe injury).

### 2.7. Determination of Pro-Inflammatory Cytokines

ELISA kit (Shanghai, China) was used to assess the level of pro-inflammatory cytokines, including IL-1β, IL-6 and TNF-α, in in vivo and in vitro samples. Mouse serum was collected from blood in each group by centrifugation. Likewise, LPS was used to induce MH-S cells to establish an inflammatory in vitro model, and the cells were subjected to COR treatment at designated doses for 24 h. Supernatant was harvested by centrifugation from individual group samples. ELISA analysis of inflammatory factors was performed.

### 2.8. Determination of mRNA Expression

Total RNA of MH-S cells and lung tissues was extracted and reverse-transcribed into cDNA Accurate Biology kit. (Changsha, China). Using a Light Cycler 480 system and the specified SYBR^®^ Green kit (Changsha, China), RT-qPCR was carried out under the following thermal cycling parameters: 95 °C for 30 s for initial denaturation, followed by 40 cycles of 95 °C for 5 s and 30 s for annealing or extension, with primer pairs listed in Table 1.

### 2.9. Determination of Key Protein in Signaling Pathway

Proteins were extracted using RIPA lysis buffer (Solarbio, R0010) supplemented with a cocktail of protease and phosphatase inhibitors (Solarbio, P1260). Following centrifugation, the supernatant was harvested, and the protein concentration was measured with a bicinchoninic acid assay kit (Shanghai, China). After quantification, protein was normalized in loading buffer. Subsequently, electrophoretic separation was performed, followed by incubation with primary antibodies and secondary antibodies, and the resulting signals were analyzed with ImageJ software (v 1.54g).

### 2.10. Statistical Analysis

All data were analyzed with SPSS (v 22.0), and plots were created using GraphPad Prism (v 9.5.1). Normality and homogeneity of variance were assessed prior to statistical testing. If the data followed a normal distribution with homogeneous variance, one-way ANOVA followed by multiple comparisons was applied. One-way ANOVA followed by multiple comparisons was applied to data exhibiting normal distribution and equal variances, whereas the Kruskal–Wallis test with Dunn’s post hoc corrections was used for non-normally distributed data. Statistical significance was defined as *p* < 0.05, with groups assigned different letters (a, b, c, d) denoting significant differences.

## 3. Results

### 3.1. Screening of Anti-Inflammatory Signaling Pathways and Targets of COR

**Figure 2:**
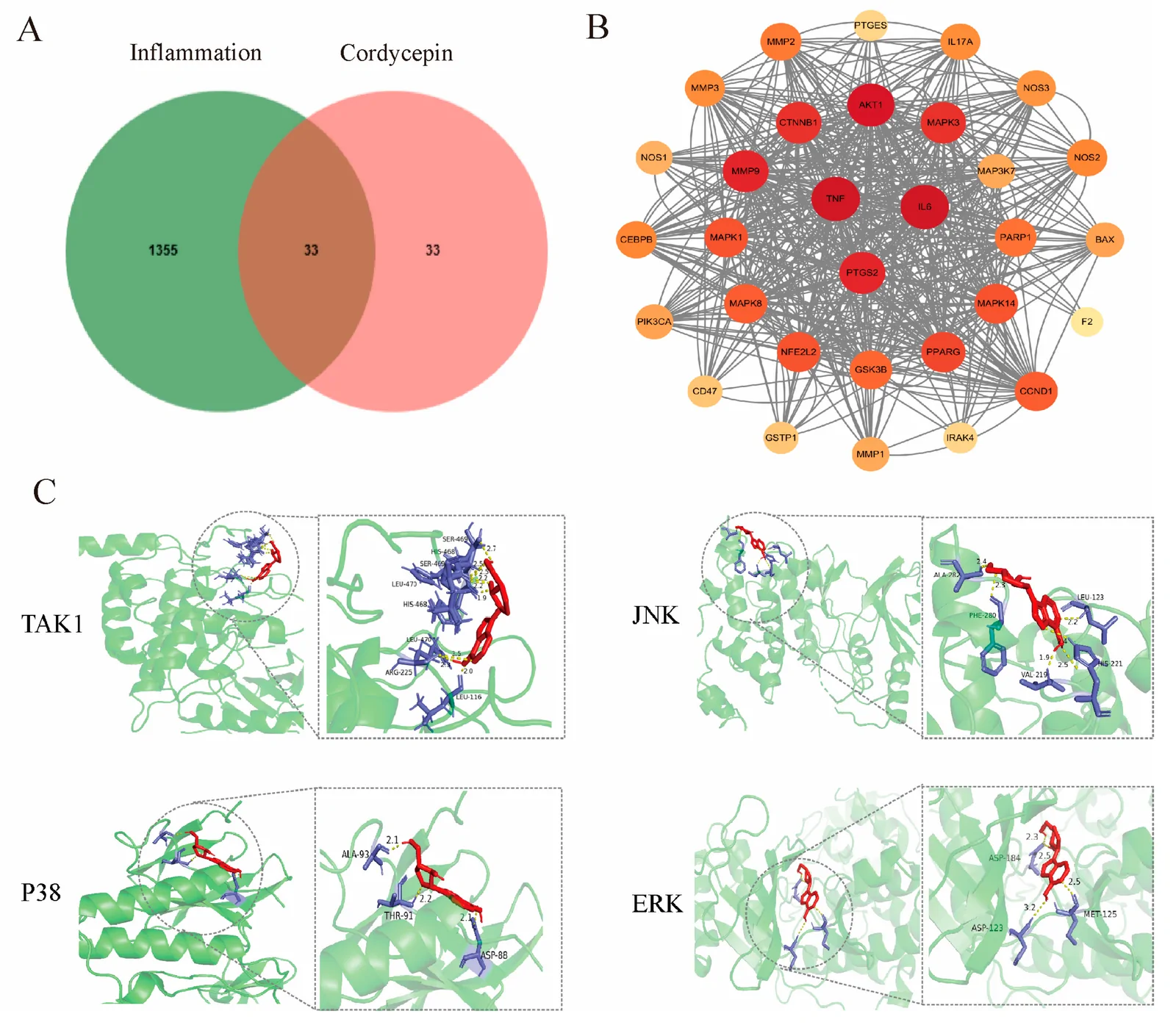
Network pharmacology and molecular docking analysis. (**A**) Overlapping targets between COR and inflammation. (**B**) The visualization of the interactions among overlapping genes, darker shades of red indicate greater importance. (**C**) Molecular docking analysis of COR with TAK1, JNK, P38 and ERK (Red: cordycepin molecules; Blue: amino acid residues; Green: proteins).

A network pharmacology approach was employed to predict targets of COR associated with inflammation. Using disease target databases, a total of 1388 inflammation-associated genes were identified, while 66 target protein genes were identified as the effective active compounds of COR based on drug target databases. Among them, 33 overlapping targets were considered potential targets for COR to treat inflammation (Figure 2A). All 33 genes in the gene set for COR treatment of inflammation were imported into STRING, and the PPI network of genes for COR treatment of inflammation was constructed by Cytoscape (Figure 2B). Based on the CytoNCA algorithm, the degree, betweenness, and closeness were screened, and the top 15 targets were selected. The key targets in PPI network were TNF, IL-6, MAPK3, MAP3K7, MMP9, NFE2L2, PPARG, MAPK1, MAPK8, GSK3B, MAPK14, PTGS2, CTNNB1 and AKT1. These targets are predicted candidate proteins participated in the anti-inflammatory effects of COR. The corresponding protein names are TNF, IL-6, ERK1, TAK1, MMP9, NRF2, NR1C3, ERK2, JNK1, GSK3B, p38α, COX-2, β-catenin, and AKT1, respectively.

To functionally characterize the 15 overlapping targets identified by network pharmacology, GO and KEGG enrichment analyses were performed. Through GO enrichment analysis, 455 enriched terms were identified, which comprised 365 biological processes (BP), 32 cellular components (CC), and 58 molecular functions (MF). The GO enrichment analysis indicates that COR may exert therapeutic effects on inflammation by inhibiting the response to LPS, inflammatory response and its regulation, MAPK cascade and its regulatory network, neuroinflammatory response regulation, kinase binding, IL-1-mediated cellular response, and protein kinase activity (Figure S1A). The KEGG pathway enrichment analysis identified 149 pathways, and the top 15 enriched pathways suggest that COR may intervene in inflammation through multiple signaling pathways such as TNF, MAPK, PI3K-Akt, NF-κB, and WNT signaling cascades, as well as other relevant pathways (Figure S1B). These results suggested that these targets were mainly associated with inflammation-related biological processes and signaling pathways, thereby providing a functional basis for subsequent key target analysis and molecular docking.

Subsequently, molecular docking analysis was conducted using Vina in the AutoDockTools (v1.5.6) software. To identify the essential binding sites and infer the binding modes between COR and these targets, 14 identified key proteins were selected for molecular docking with COR. In molecular docking, a greater number of hydrogen bonds and a lower binding energy indicate stronger interactions between ligand and receptor, as well as more stable binding. Binding energies of −5.227 kcal/mol or less denote good ligand and receptor affinity [36]. The results showed that TAK1, JNK1, p38α and ERK1 exhibit good binding activity with COR. Among them, TAK1 has the most hydrogen bonds with COR, and the binding is predominantly stabilized by multiple hydrogen bonds and hydrophobic interactions with its proximal amino acid residues. In addition, TAK1 has the lowest binding free energy with COR, at —6.42 kcal/mol (Figure 2C). Therefore, COR exhibits high-affinity binding to multi-target proteins and the most stable binding with TAK1. We next probed whether TAK1 mediates the simultaneous inhibition of the MAPK and NF-κB pathways by COR.

### 3.2. COR Mitigates LPS-Induced Inflammation by Inhibition of MAPK Pathway by Targeting TAK1

**Figure 3:**
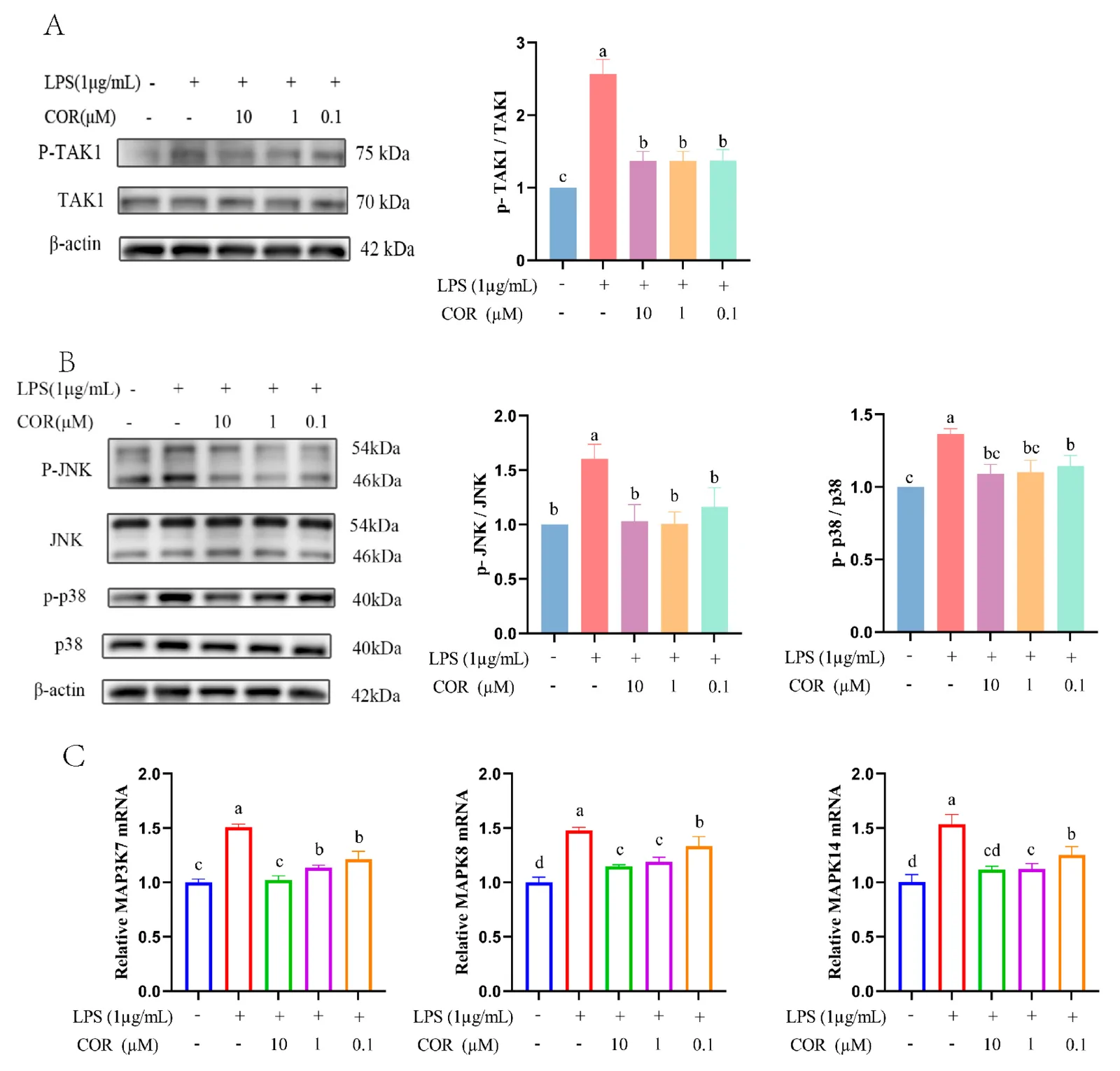
COR inhibits the MAPK pathway in MH-S cells. (**A**,**B**) WB analysis of P-TAK1/TAK1, p-p38/p38, p-JNK/JNK levels in the MH-S cells treated with LPS and 10 µM, 1 µM, and 0.1 µM of COR over 48 h (*n* = 3). (**C**) RT-qPCR analysis of MAP3K7, MAPK14 and MAPK8 mRNA levels in the MH-S cells treated with LPS and 10 µM, 1 µM, and 0.1 µM of COR over 48 h (*n* = 9). Data are shown as mean ± SD. Groups that share the same letter are not significantly different from each other (*p* ≥ 0.05), while groups with different letters differ significantly (*p* < 0.05). COR, cordycepin; LPS, lipopolysaccharide; TAK1, TGF-β-activated kinase 1; WB, Western blot. The original western blot images can be found in Supplementary Materials.

The P-TAK1/TAK1 protein ratio was markedly elevated in LPS-induced MH-S cells, while COR had a significant inhibitory effect on this increasing trend (*p* < 0.05) (Figure 3A). Meanwhile, the JNK and P38 phosphorylation levels were significantly upregulated, and these changes were reversed by COR treatment (Figure 3B). In addition, LPS significantly elevated the mRNA relative expression of MAP3K7, MAPK14 and MAPK8 in MH-S cells. However, after being treated with COR, these elevated mRNA levels were markedly inhibited (*p* < 0.05) (Figure 3C). These results show that COR markedly downregulates and effectively mitigates the protein expression and then the mRNA levels of key factors.

**Figure 4:**
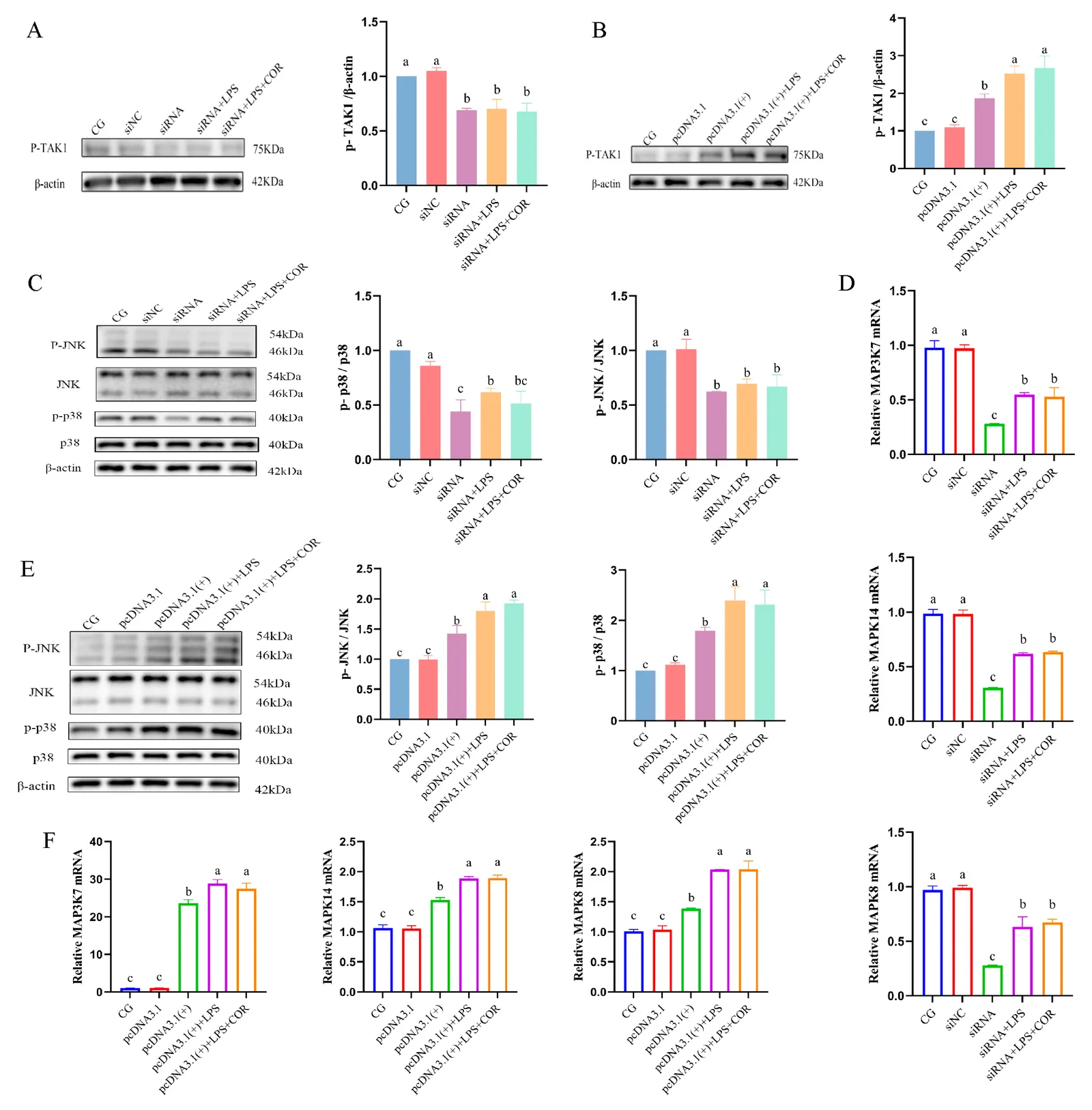
The suppressive effect of COR on LPS-induced MAPK activation was abrogated by either TAK1 knockdown or its overexpression. (**A**,**C**) WB analysis of P-TAK1, p-p38/p38, and p-JNK/JNK levels with knockdown of TAK1 in MH-S cells challenged with LPS and 1 µM of COR over 54 h (*n* = 3). (**D**) RT-qPCR analysis for MAP3K7, MAPK14 and MAPK8 mRNA levels with knockdown of TAK1 in MH-S cells challenged with LPS and 1 µM of COR over 54 h (*n* = 9). (**B**,**E**) WB analysis of P-TAK1, p-p38/p38, and p-JNK/JNK levels with overexpression of TAK1 in MH-S cells challenged with LPS and 1 µM of COR over 54 h (*n* = 3). (**F**) RT-qPCR analysis of MAP3K7, MAPK14 and MAPK8 mRNA levels with overexpression of TAK1 in MH-S cells treated with LPS and 1 µM of COR over 54 h (*n* = 9). Data are shown as mean ± SD. Groups that share the same letter are not significantly different from each other (*p* ≥ 0.05), while groups with different letters differ significantly (*p* < 0.05). COR, cordycepin; LPS, lipopolysaccharide; TAK1, TGF-β-activated kinase 1; CG, control group; siNC, siRNA negative control. The original western blot images can be found in Supplementary Materials.

To assess whether COR alleviates LPS-induced MH-S cell inflammation via targeting and regulating the TAK1-MAPK signaling pathway, we performed TAK1 knockdown and overexpression in MH-S cells. The TAK1, p38 and JNK phosphorylation levels were significantly decreased with knockdown of TAK1 in MH-S cells challenged with LPS, while treatment with COR was unable to increase the expression of proteins in MH-S cells challenged with LPS (*p* < 0.05) (Figure 4A,C). Similarly, this knockdown notably decreased the relative mRNA expression of MAP3K7, MAPK8, and MAPK14 in MH-S cells challenged with LPS (*p* < 0.05). However, COR failed to exert a therapeutic effect in LPS-stimulated MH-S cells following TAK1 knockdown, indicating that silencing TAK1 abolished the anti-inflammatory action of COR (Figure 4D).

Nevertheless, the TAK1, p38 and JNK phosphorylation levels were markedly increased with overexpression of TAK1 in MH-S cells challenged with LPS (*p* < 0.05). Likewise, treatment with COR failed to reduce the expression of these proteins (Figure 4B,E). LPS significantly increased the mRNA relative expression of MAP3K7, MAPK8, and MAPK14 in MH-S cells with overexpression of TAK1; treatment with COR could no longer suppress the mRNA relative expression of MAP3K7, MAPK8, and MAPK14 in MH-S cells (*p* < 0.05) (Figure 4F). These results indicate that the protective effects of COR were compromised by TAK1 overexpression in MH-S cells. These data demonstrate that the anti-inflammatory activity of COR on LPS-challenged MH-S cells is attenuated under conditions of either TAK1 deficiency or TAK1 overexpression. This further indicates that the anti-inflammatory effect of COR is closely associated with TAK1-dependent signaling.

### 3.3. COR Mitigates LPS-Induced Inflammation by Inhibition of the NF-κB Pathway by Targeting TAK1

**Figure 5:**
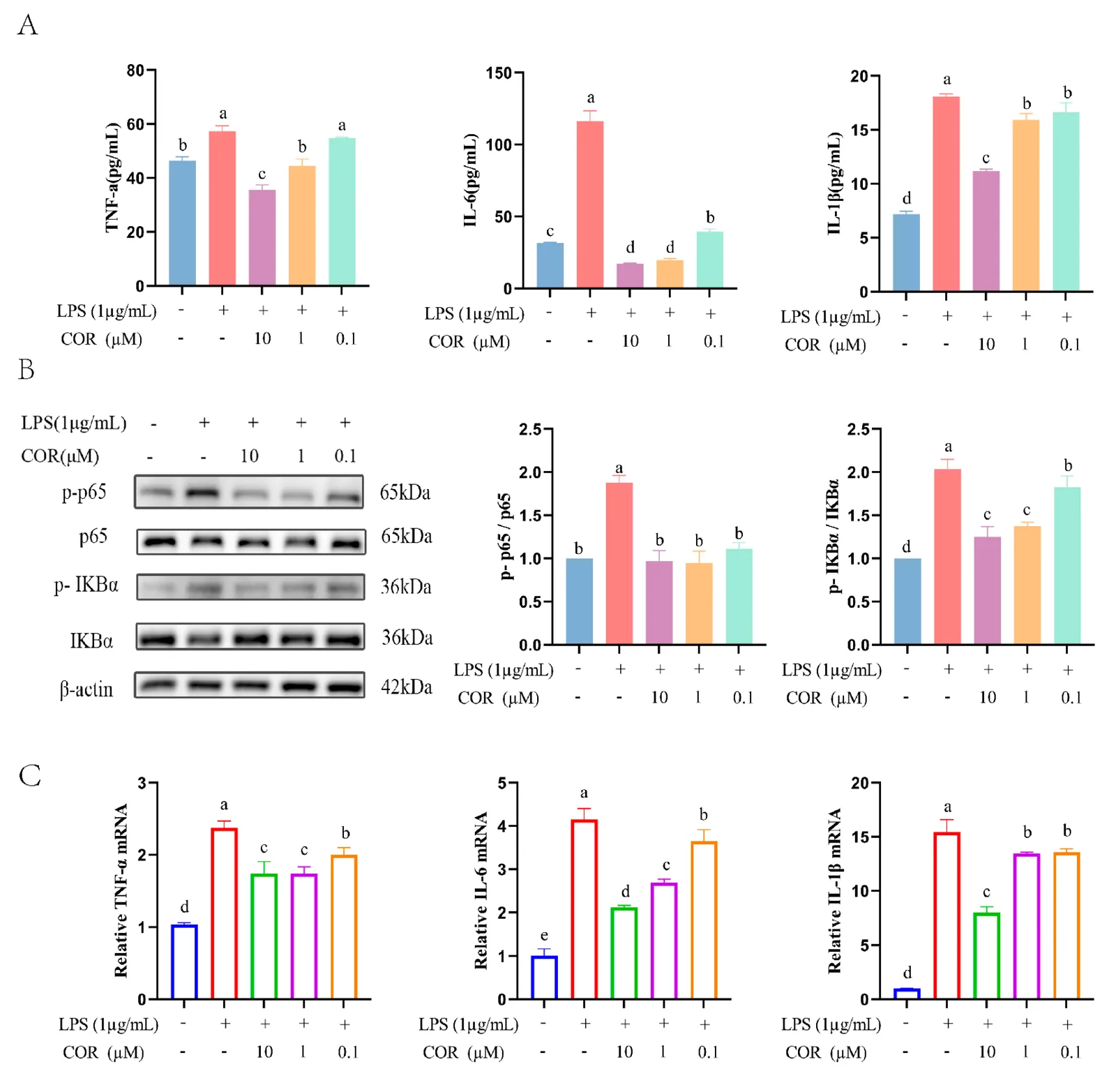
COR inhibits the NF-κB pathway in MH-S cells. (**A**) ELISA analysis of TNF-α, IL-6 and IL-1β protein levels in the MH-S cell culture supernatant treated with LPS and 10 µM, 1 µM, and 0.1 µM of COR over 48 h (*n* = 6). (**B**) WB analysis of P-IκB-α/IκB-α and p-p65/p65 levels in MH-S cells challenged with LPS and 10 µM, 1 µM, and 0.1 µM of COR over 48 h (*n* = 3). (**C**) RT-qPCR analysis of TNF-α, IL-6, and IL-1β mRNA levels in the MH-S cells treated with LPS and 10 µM, 1 µM, and 0.1 µM of COR over 48 h (*n* = 9). Groups that share the same letter are not significantly different from each other (*p* ≥ 0.05), while groups with different letters differ significantly (*p* < 0.05). COR, cordycepin; LPS, lipopolysaccharide; TAK1, TGF-β-activated kinase 1. The original western blot images can be found in Supplementary Materials.

The supernatant levels of TNF-α, IL-6, and IL-1β were significantly elevated in LPS-stimulated MH-S cells compared with the CG (*p* < 0.05). Compared with the MG, the TNF-α and IL-1β protein levels in MH-S cells challenged with LPS were significantly decreased after being treated with COR at 1 µM and 10 µM (*p* < 0.05), while the IL-6 protein level was markedly decreased at 10 µM, 1 µM and 0.1 µM COR compared to the MG (*p* < 0.05) (Figure 5A). Subsequent LPS exposure significantly enhanced the phosphorylation of IκB-α and P65 in MH-S cells (*p* < 0.05). The protein levels of these targets were markedly downregulated by COR treatment (Figure 5B). In addition, LPS stimulation significantly upregulated the TNF-α, IL-6, and IL-1β mRNA levels in MH-S cells (*p* < 0.05). However, COR markedly inhibited these elevated mRNA levels (Figure 5C). These findings indicate that COR markedly downregulates critical proteins of the NF-κB pathway, thus effectively attenuating LPS-induced inflammation.

**Figure 6:**
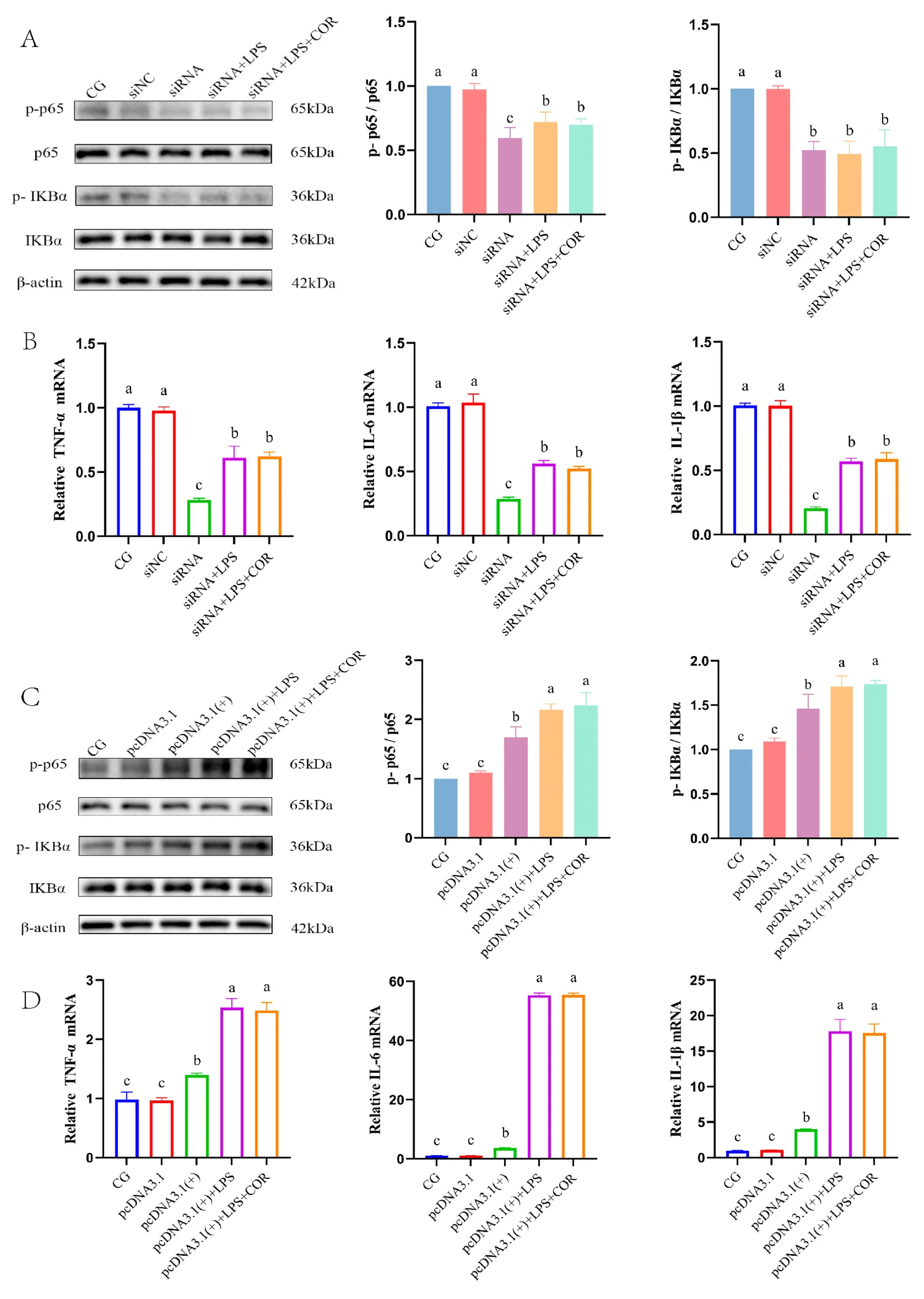
TAK1 knockdown and overexpression eliminate the suppressive impact of COR on LPS-induced NF-κB activation. (**A**) WB analysis of P-IκB-α/IκB-α and p-p65/p65 levels with knockdown of TAK1 in MH-S cells challenged with LPS and 1 µM of COR over 54 h (*n* = 3). (**B**) RT-qPCR analysis of TNF-α, IL-6, and IL-1β mRNA levels with knockdown of TAK1 in MH-S cells challenged with LPS and 1 µM of COR over 54 h (*n* = 9). (**C**) WB analysis of P-IκB-α/IκB-α and p-p65/p65 levels with overexpression of TAK1 in MH-S cells challenged with LPS and 1 µM of COR over 54 h (*n* = 3). (**D**) RT-qPCR analysis of TNF-α, IL-6, and IL-1β mRNA levels with overexpression of TAK1 in MH-S cells challenged with LPS and 1 µM of COR over 54 h (*n* = 9). Data are shown as mean ± SD. Groups that share the same letter are not significantly different from each other (*p* ≥ 0.05), while groups with different letters differ significantly (*p* < 0.05). COR, cordycepin; LPS, lipopolysaccharide; TAK1, TGF-β-activated kinase 1; CG, control group; siNC, siRNA negative control. The original western blot images can be found in Supplementary Materials.

To further assess whether COR alleviates MH-S cells challenged with LPS-induced inflammation by targeting and regulating the TAK1-NF-κB signaling pathway, we also constructed TAK1 knockdown and overexpression in MH-S cells. The phosphorylation levels of IκB-α and P65 in MH-S cells challenged with LPS were significantly reduced with TAK1 knockdown, while treatment with COR was unable to increase the expression of proteins in LPS-induced MH-S cells (*p* < 0.05) (Figure 6A). This knockdown notably decreased TNF-α, IL-6 and IL-1β in MH-S cells challenged with LPS (*p* < 0.05). Nevertheless, COR failed to exert anti-inflammatory activity in LPS-challenged MH-S cells subjected to TAK1 knockdown, revealing that silencing TAK1 counteracted COR’s suppressive action (Figure 6B). However, the phosphorylation level of IκB-α and P65 in MH-S cells challenged with LPS was significantly increased with overexpression of TAK1 (*p* < 0.05). Subsequent COR treatment failed to downregulate the expression of these proteins, suggesting that TAK1 overexpression dampened COR’s suppressive effects on MH-S cell inflammation (Figure 6C). Although LPS significantly increased the mRNA relative expression of TNF-α, IL-6 and IL-1β in MH-S cells with overexpression of TAK1 (*p* < 0.05), subsequent treatment with 1 μM COR failed to inhibit the TNF-α, IL-6, and IL-1β mRNA relative expressions in MH-S cells (Figure 6D), revealing that TAK1 overexpression counteracted COR’s anti-inflammatory action.

### 3.4. COR Attenuates LPS-Induced ALI in Mice

**Figure 7:**
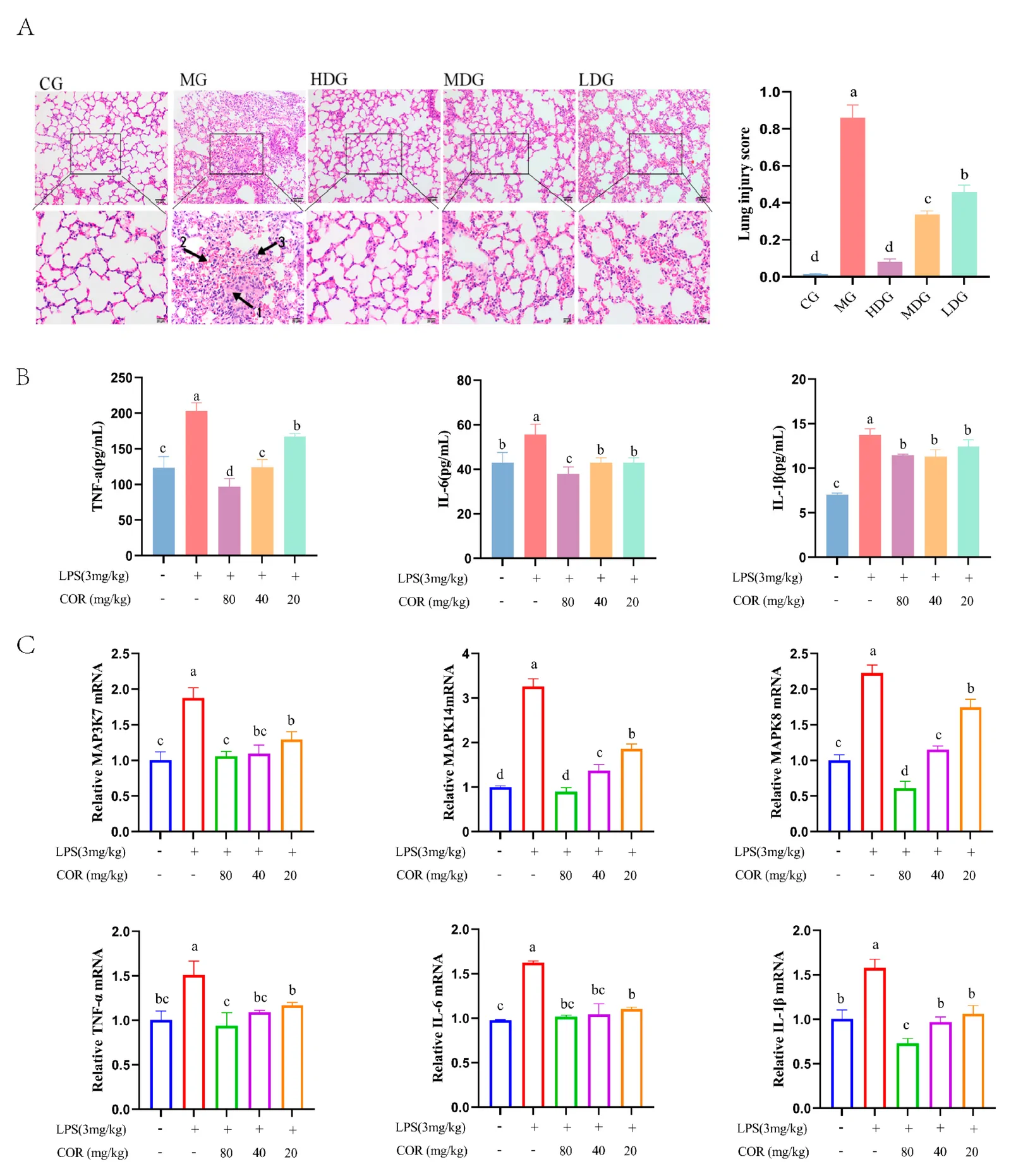
COR ameliorated LPS-induced ALI in mice. (**A**) H&E staining of pulmonary sections followed by lung injury scoring; black arrow 1, fibrinous exudation; black arrow 2, alveolar septal hyperemia; black arrow 3, neutrophilic infiltration. (**B**) ELISA analysis of TNF-α, IL-6 and IL-1β protein levels in mouse serum treated with LPS and 80 mg/kg, 40 mg/kg, and 20 mg/kg of COR over 4 days (*n* = 6). (**C**) RT-qPCR analysis of MAP3K7, MAPK14, MAPK8, TNF-α, IL-6 and IL-1β mRNA levels in mouse lung tissues treated with LPS and 80 mg/kg, 40 mg/kg, and 20 mg/kg of COR over 4 days (*n* = 9). Data are shown as mean ± SD. Groups that share the same letter are not significantly different from each other (*p* ≥ 0.05), while groups with different letters differ significantly (*p* < 0.05). The groups are represented as follows: CG, control group; MG, LPS group; HDG, MG + 80 mg/kg COR; MDG, MG + 40 mg/kg COR; LDG, MG + 20 mg/kg COR. COR, cordycepin; LPS, lipopolysaccharide; ALI, acute lung injury; H&E, hematoxylin–eosin.

The alveolar morphology of lungs in the CG was regularly arranged and closely connected, and exhibited an intact cellular structure, with no hemorrhage or exudation. Conversely, alveolar structural injury was noted in the lung tissues of the MG group, with cellular exudation, hemorrhage, inflammation in the interstitial spaces, and shedding of ciliated columnar epithelium in the lumen. Compared to MG, intervention with different concentrations of COR improved the pulmonary alveolar structure, significantly decreased the number of cells, and alleviated bleeding and congestion. Similarly, LPS challenge led to a marked elevation in lung injury scores. This increase was significantly attenuated by COR treatment at all tested doses (*p* < 0.05), with the HDG exhibiting the most pronounced improvement (Figure 7A).

The TNF-α, IL-6 and IL-1β protein contents were markedly increased in the serum from the inflamed mice instilled with LPS (*p* < 0.05). After being treated with COR at concentrations of 20, 40, and 80 mg/kg, TNF-α and IL-6 protein levels were markedly reduced. However, the protein level of IL-1β was not significantly reduced at 20 mg/kg of COR (*p* < 0.05) (Figure 7B). As well as the mRNA expressions of MAP3K7, MAPK14, MAPK8, TNF-α, IL-6 and IL-1β was markedly increased in LPS-induced mouse lungs. However, COR markedly inhibited these elevated mRNA levels (Figure 7C) (*p* < 0.05). The results indicate that the administration of various concentrations of COR markedly alleviated LPS-induced ALI in mice.

### 3.5. COR Mitigates LPS-Induced Inflammation by Inhibition of TAK1-MAPK and NF-κB Pathway In Vivo

**Figure 8:**
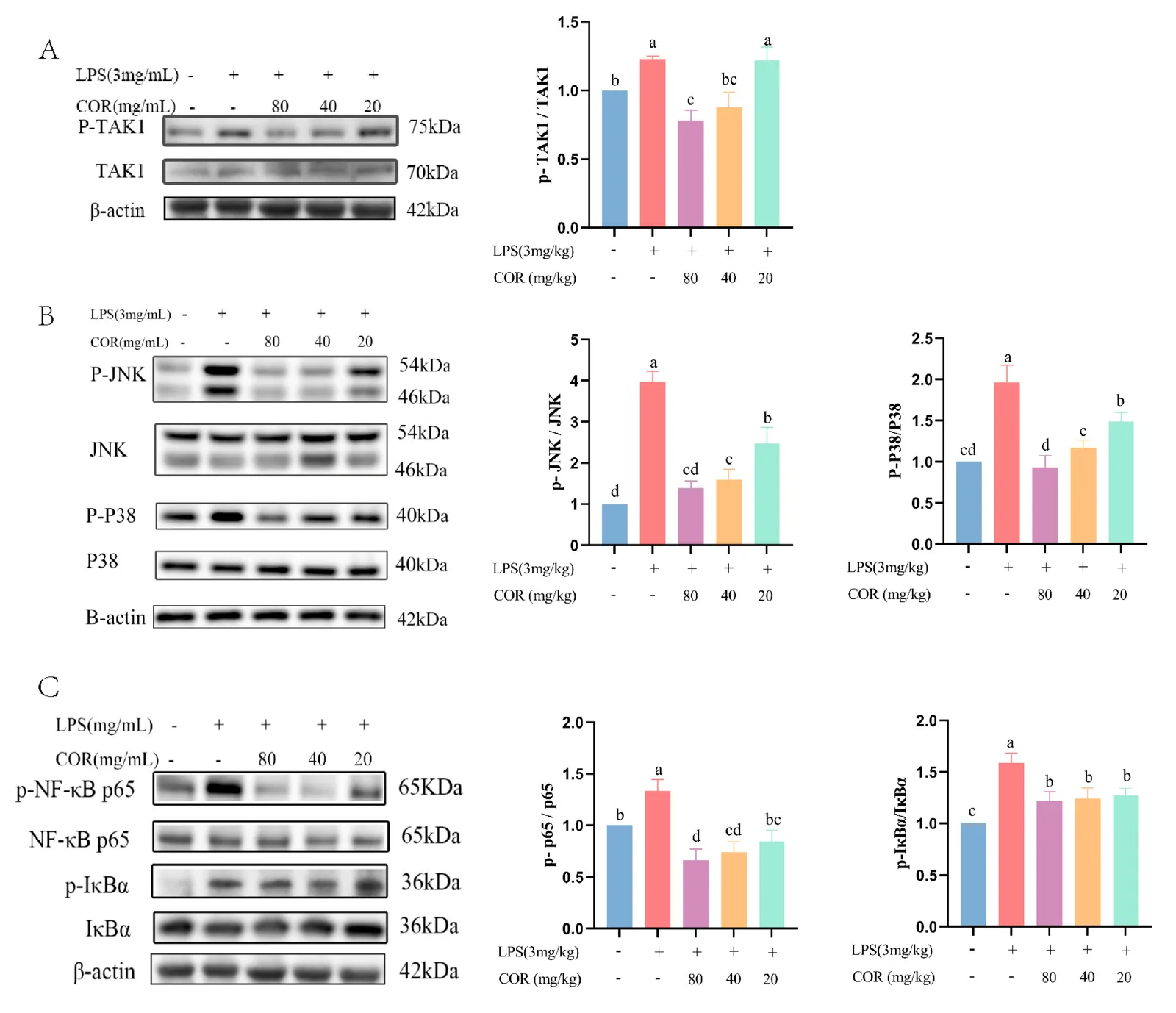
WB analyses of key protein expression in the MAPK and NF-κB signaling pathways in vivo. (**A**–**C**) WB analysis of P-TAK1/TAK1, p-p38/p38, p-JNK/JNK, p-P65/P65 and p-IκB-α/IκB-α protein levels in mouse serum treated with LPS and 80 mg/kg, 40 mg/kg, and 20 mg/kg of COR over 4 days (*n* = 3). Data are shown as mean ± SD. Groups that share the same letter are not significantly different from each other (*p* ≥ 0.05), while groups with different letters differ significantly (*p* < 0.05). COR, cordycepin; LPS, lipopolysaccharide; WB, Western blot. The original western blot images can be found in Supplementary Materials.

In the in vivo test, the protein expression of P-TAK1/TAK1, P-JNK/JNK and P-P38/P38 was significantly upregulated in the lungs of mice instilled intranasally with LPS. Treatment with COR markedly attenuated the phosphorylation levels of these proteins (Figure 8A,B) (*p* < 0.05). These data demonstrated that COR could possibly inhibit ALI in mice via MAPK pathway regulation.

Similarly, significantly increased ratios of P-IκB-α/IκB-α and P-P65/P65 were observed in the lung tissues of LPS-treated mice, which were reduced upon COR administration (Figure 8C). In summary, these results are consistent with those obtained in vitro. This indicates that COR alleviates ALI induced by LPS in vivo by inhibiting the TAK1-MAPK and NF-κB pathways.

## 4. Discussion

ALI represents a severe and rapidly progressive form of pulmonary inflammation defined by acute onset, often culminating in acute respiratory distress syndrome with substantial clinical burden. Inflammation serves as a crucial defensive response that mediates innate host defense against tissue damage and infection [37,38]. Specifically, numerous immune cells infiltrate infection sites and activate multiple inflammatory signaling cascades to secrete abundant inflammatory mediators [39]. If the exposure to pro-inflammatory cytokines cannot be controlled by the body’s innate physiological mechanism, this inflammation subsequently impairs the alveolar epithelium and vascular endothelial barriers and increases alveolar-capillary permeability. As a result, proteinaceous edema permeates the alveolar walls and lumina, instigating septal hypertrophy and consequential damage to the pulmonary architecture [40,41]. COR, a natural nucleoside analog extracted from *Cordyceps militaris*, possesses diverse bioactivities [42]. Given its multi-component and multi-target characteristics, its therapeutic effects cannot be fully elucidated by examining a single signaling pathway. Network pharmacology addresses this limitation by providing a systematic framework to predict bioactive compounds, and it conforms to the holistic philosophy of TCM [43,44]. By integrating network pharmacology, molecular docking, and experimental validation, this study elucidates the active components and anti-inflammatory mechanisms of COR, underscoring its promise as a viable candidate for clinical translation.

Network pharmacology, which combines bioinformatics and polypharmacology, is emerging as a promising and cost-effective approach for drug discovery [45]. To elucidate the molecular mechanisms, we first predicted the critical targets of COR for inflammation treatment through network pharmacology. The critical targets of COR for inflammation treatment were identified, including inflammatory factors TNF-α, IL-6, MAP3K7, MAPK8 and MAPK14. Furthermore, network pharmacology-based enrichment analysis showed that COR is associated with key biological processes such as the LPS-mediated signaling pathway, stress-activated MAPK cascade, and inflammatory response, as well as critical pathways identified using this method. It is well documented that the MAPK pathway mediates its signaling via phosphorylation of JNK and p38, which subsequently triggers the activation of nuclear transcription factors [46,47,48]. Moreover, a close interplay exists between the MAPK and NF-κB pathways, whereby the MAPK pathway regulates inflammatory cytokine expression by activating NF-κB; however, NF-κB activation involves the phosphorylation and degradation of IκB-α, leading to dissociation of the NF-κB/IκB-α complex [49]. This enables p65 to migrate from the cytoplasm to the nucleus, leading to an increase in inflammatory cytokine production, culminating in the transcriptional modulation of both cytokines and ancillary inflammatory signals [50]. The activated NF-κB and MAPK pathways in the cytoplasm regulate the expression of various inflammatory mediators such as TNF-α, IL-1β, and IL-6 [51]. It has been previously documented that alveolar macrophages can trigger immune activation and inflammatory responses through phagocytosis of foreign particles, playing a pivotal role in respiratory immune defense mechanisms [52]. Given the pivotal role of the MAPK and NF-κB signaling pathways in modulating inflammation, it was prioritized for experimental validation. The effective binding of COR to TAK1, JNK1, p38α, and ERK1 was revealed by molecular docking, providing evidence for a multi-target anti-inflammatory mechanism and further substantiating the network pharmacology-based predictions. Notably, TAK1 exhibited the highest binding energy among these targets. Similar to these findings, our results revealed that COR directly attenuates the phosphorylation of TAK1, the pivotal upstream regulator shared by these signaling routes, and the expression levels of key proteins in the MAPK and NF-κB pathways in LPS-induced MH-S, as well as inflammation factor levels and the relative mRNA expression of these key targets. These findings suggest that the anti-inflammatory effect of COR was found to be mediated through inhibition of the MAPK and NF-κB pathways.

As a serine and threonine kinase, TAK1 represents a prominent member of the MAPK family [53]. Full activation of TAK1, characterized by phosphorylation at Ser192 and Thr184/187, is triggered by a range of stimuli, including cytokines, pathogens, LPS, hypoxia, and DNA damage [54,55]. Consequently, the activation of TAK1 leads to phosphorylation of downstream substrates, which in turn initiates NF-κB and MAPK signaling, with subsequent involvement in inflammation, immunity, fibrosis, cell death, and malignant progression [56,57]. Subsequently, to further validate the causal relationship between COR and the TAK1/MAPK and NF-κB pathways, MH-S cell models featuring TAK1 knockdown or overexpression were constructed to probe the regulatory function of TAK1 in inflammation. Knockdown of TAK1 resulted in reduced phosphorylation of TAK1, p38, JNK, IκB-α, and p65, and accordingly, decreased transcription of TNF-α, IL-6, IL-1β, MAP3K7, MAPK8, and MAPK14. However, COR failed to demonstrate therapeutic efficacy in LPS-stimulated MH-S cells in the context of TAK1 knockdown. In contrast, TAK1 overexpression led to significantly increased TAK1, p38, JNK, IκB-α and P65 phosphorylation levels, as well as increased TNF-α, IL-6, IL-1β, MAP3K7, MAPK8 and MAPK14 relative expression. Similarly, TAK1 overexpression counteracted the inhibitory effects of COR on inflammation response. The present data unequivocally link TAK1 abundance to the activation status of inflammatory cascades, underscoring its pivotal role in immune signaling. Therefore, the mechanism by which COR suppresses inflammation response could target TAK1, affecting the downstream activation of the MAPK and NF-κB pathways, and further repressing the activation of inflammation-related genes.

To verify the in vitro results, we therefore carried out an in vivo examination to investigate the anti-inflammatory effect of COR. The results showed that COR 40 mg/kg had a markedly inhibitory effect in ALI mice, while lower concentrations were less effective. The suppressive effect of COR was on the MAPK and NF-κB pathways; it reduced the phosphorylation of TAK1, p38, JNK, IκB-α and P65, and then reduced the production of TNF-α, IL-6, and IL-1β, as well as the relative mRNA expression of MAP3K7, MAPK8 and MAPK14, which were consistent with the in vitro findings.

In summary, our systematic assessment elucidates the anti-inflammatory actions and cognate molecular targets of COR, supporting its potential as a therapeutic agent for ALI. Nevertheless, the translation of these preclinical findings into clinical application remains constrained by several unresolved challenges. From a pharmacological perspective, in vivo efficacy is limited not merely by pharmacokinetic properties, but more importantly by the absence of systematic assessments of tissue-specific efficacy and drug interaction risks within complex in vivo inflammatory settings, factors that together hinder clinical translation [58]. Moreover, the safety profile of COR, including long-term tolerability, dose optimization, and tissue-specific toxicity, warrants more comprehensive toxicological characterization [59]. Importantly, the current evidence base remains largely confined to in vitro and animal models, with robust clinical data on efficacy and safety in humans still lacking. Therefore, while COR emerges as a compelling lead compound for inflammatory pulmonary diseases, its advancement as a viable therapeutic strategy will require concerted efforts in pharmacological optimization, toxicological evaluation, and well-designed translational and clinical studies.

## 5. Conclusions

In conclusion, COR exerts a therapeutic effect on acute lung injury by directly targeting TAK1 to inhibit the activation of the MAPK and NF-κB signaling pathways and reducing the expression of key inflammatory cytokines. These findings not only elucidate the molecular mechanism underlying the therapeutic action of COR in ALI but also provide a promising candidate for clinical intervention in inflammatory respiratory diseases.

## Figures and Tables

**Table 1:** The primers used in RT-qPCR.

Gene	Forward Primer (5′—3′)	Reverse Primer (5′—3′)	Size (bp)	Gene Bank Accession
GAPDH	GCGGCATTCACGAAACTACCTT	TCCTGCTIGCTGATCCACATCT	268	NM 173979.3.
IL-1β	GCAGGCAGTATCACTCATTGT	GGCTTTTTTGTTGTTCATCTC	221	NM_008361.4
IL-6	GTTCTCTGGGAAATCGTGGA	GCATTGGAAATTGGGCTAGG	344	NM_031168.2
TNF-α	AAGGGAGAGTGGTCAGGTTGG	CAGAGGTTCAGTGATGTAGCG	415	NM_013693.3
MAP3K7	CGAGGTGGAAGAGGTTGTCG	TCAGGCAGGCTCCATACAAC	102	NM_145331.3
MAPK8	GTGGAATCAAGCACCTTCACT	TCCTCGCCAGTCCAAAATCAA	114	XM_021203479.2
MAPK14	AGGAGAGGCCCACGTTCTAC	CAAAAGCAGCACACACCGA	120	NM_001168508.1

## Data Availability

The original contributions presented in this study are included in the article. Further inquiries can be directed to the corresponding authors.

## Supplementary Materials

The following supporting information can be downloaded at: https://www.mdpi.com/article/10.3390/biom16091279/s1, Figure S1: GO and KEGG enrichment analysis; Supplementary Material S2: Original WB images.

## Abbreviations

The following abbreviations are used in this manuscript:

ALI	Acute lung injury
TCM	Traditional Chinese medicine
COR	Cordycepin
LPS	Lipopolysaccharide
TAK1	TGF-β-activated kinase 1
MAPK	Mitogen-activated protein kinase
NF-κB	Nuclear factor-κB
GO	Gene ontology
KEGG	Kyoto encyclopedia of genes and genomes
BP	Biological processes
MF	Molecular functions
CC	Cellular components
PPI	Protein–protein interaction
FBS	Fetal bovine serum
CG	Control group
MG	Model group
HDG	High dose group
MDG	Middle dose group
LDG	Low dose group
HE	Hematoxylin–Eosin Staining
PVDF	Polyvinylidene fluoride
