# Supplementary material for: Cordycepin Alleviates Acute Lung Injury by Targeting TAK1 to Inhibit MAPK and NF-κB Signaling Pathways
Source: Biomolecules. 2026 Sep 3;16(9):1279. doi: 10.3390/biom16091279 (PMC13604512; doi:10.3390/biom16091279)
Supplement: Supplementary file 1 [file biomolecules-16-01279-s001.zip › supplementary material S2-original WB figure/biomolecules-4411057-original WB figures.pdf]

Figure 3 Original western blot for three repeats

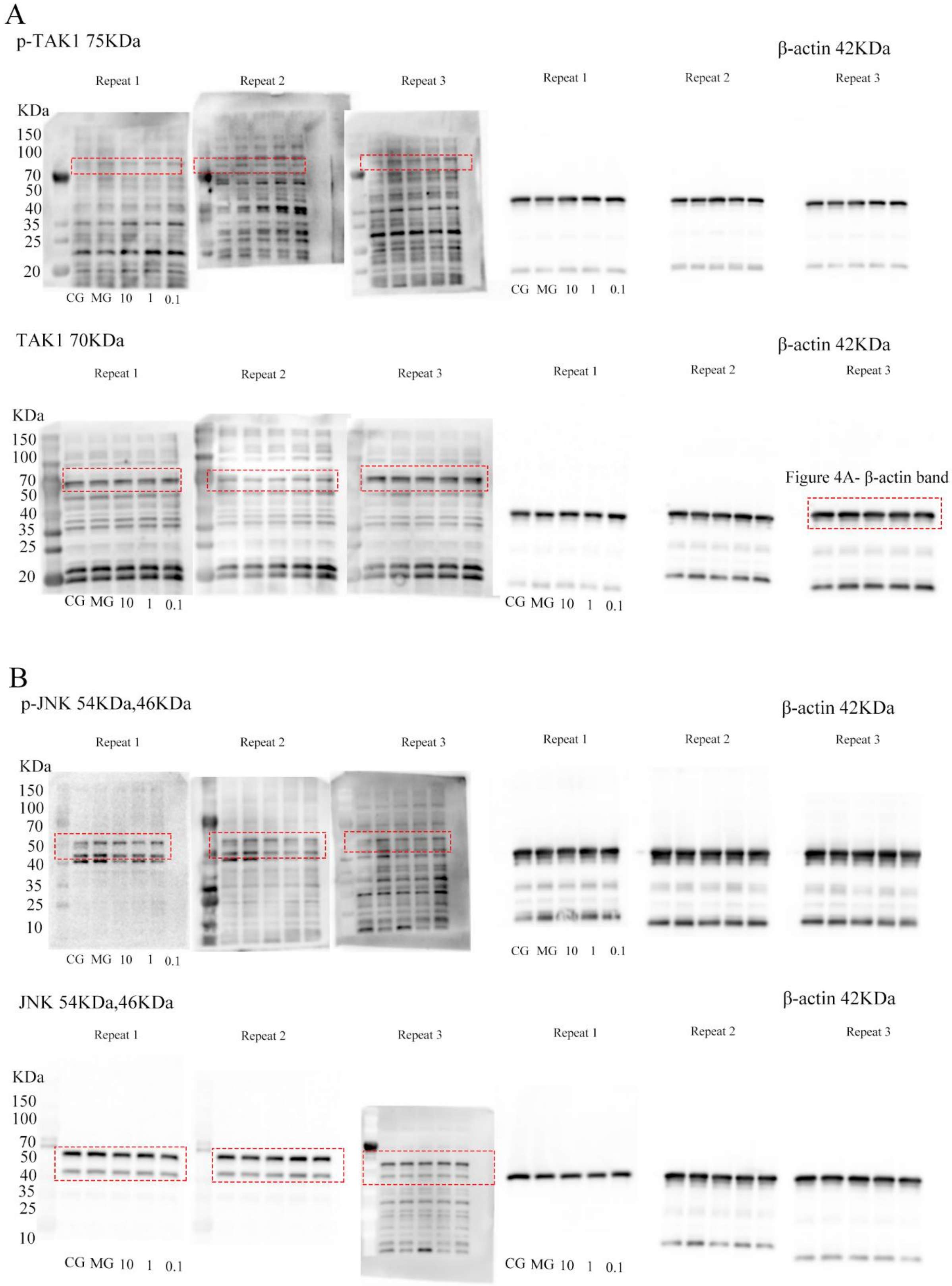

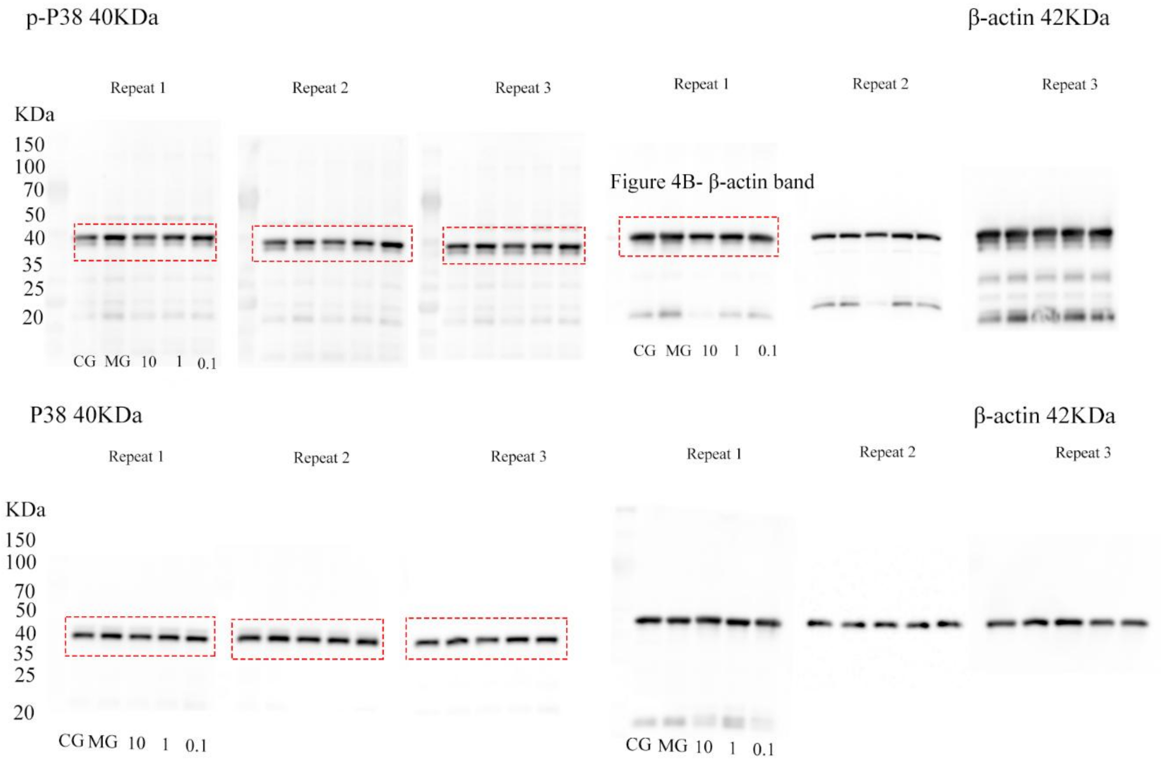

Figure 4 Original western blot for three repeats

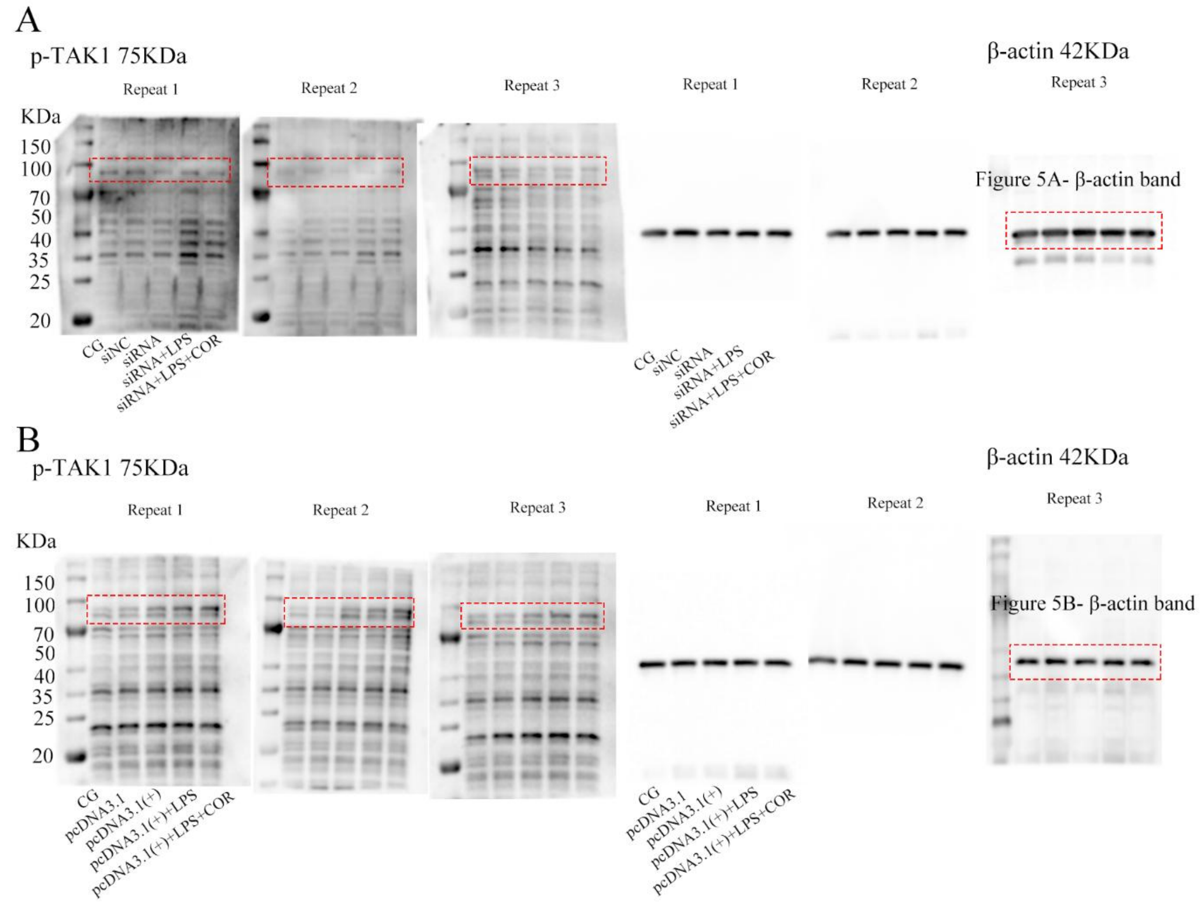

C

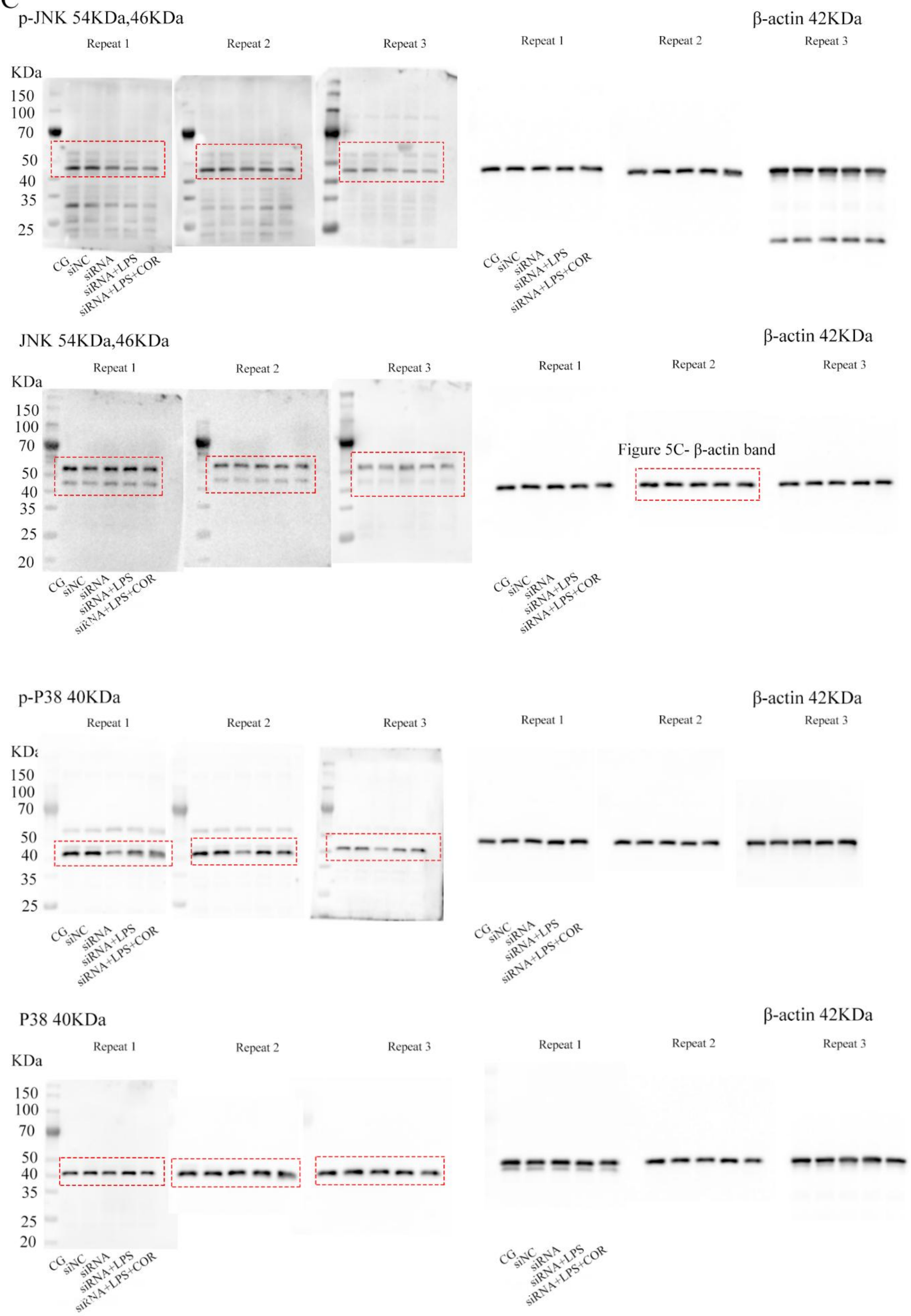
p-JNK 54KDa,46KDa
β-actin 42KDa
Repeat 1
Repeat 2
Repeat 3
KDa
150
100
70
50
40
35
25
CG
siNC
siRNA
siRNA+LPS
siRNA+LPS+COR
JNK 54KDa,46KDa
β-actin 42KDa
Repeat 1
Repeat 2
Repeat 3
KDa
150
100
70
50
40
35
25
20
Figure 5C- β-actin band
CG
siNC
siRNA
siRNA+LPS
siRNA+LPS+COR
p-P38 40KDa
β-actin 42KDa
Repeat 1
Repeat 2
Repeat 3
KDa
150
100
70
50
40
35
25
CG
siNC
siRNA
siRNA+LPS
siRNA+LPS+COR
P38 40KDa
β-actin 42KDa
Repeat 1
Repeat 2
Repeat 3
KDa
150
100
70
50
40
35
25
20
CG
siNC
siRNA
siRNA+LPS
siRNA+LPS+COR

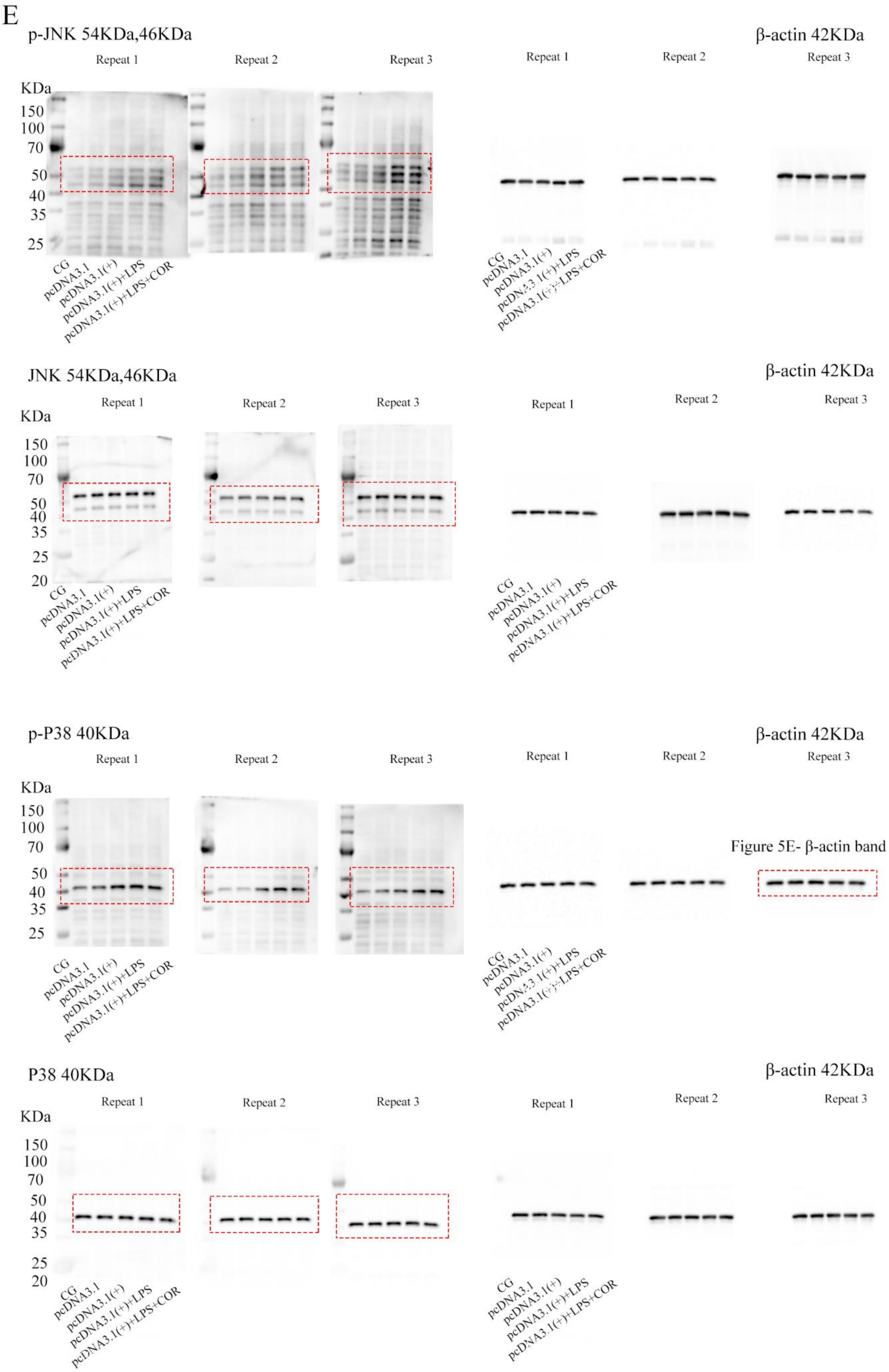
E
p-JNK 54KDa,46KDa
β-actin 42KDa
Repeat 1
Repeat 2
Repeat 3
KDa
150
100
70
50
40
35
25
CG
pcDNA3.1
pcDNA3.1(+)
pcDNA3.1(+)+LPS
pcDNA3.1(+)+LPS+COR
JNK 54KDa,46KDa
β-actin 42KDa
20
p-P38 40KDa
β-actin 42KDa
Figure 5E- β-actin band
P38 40KDa
β-actin 42KDa

Figure 5 Original western blot for three repeats

B

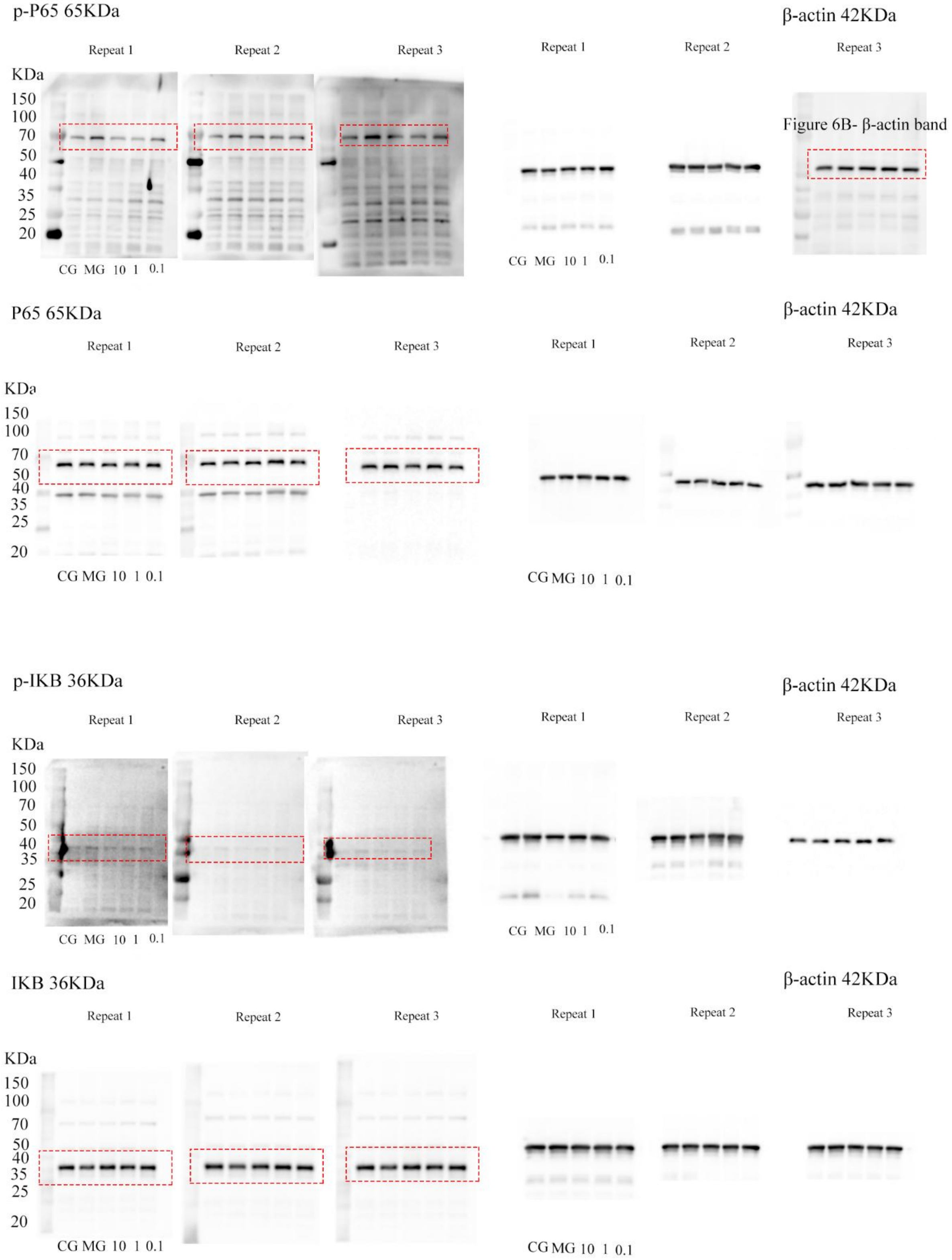

Figure 6 Original western blot for three repeats

A

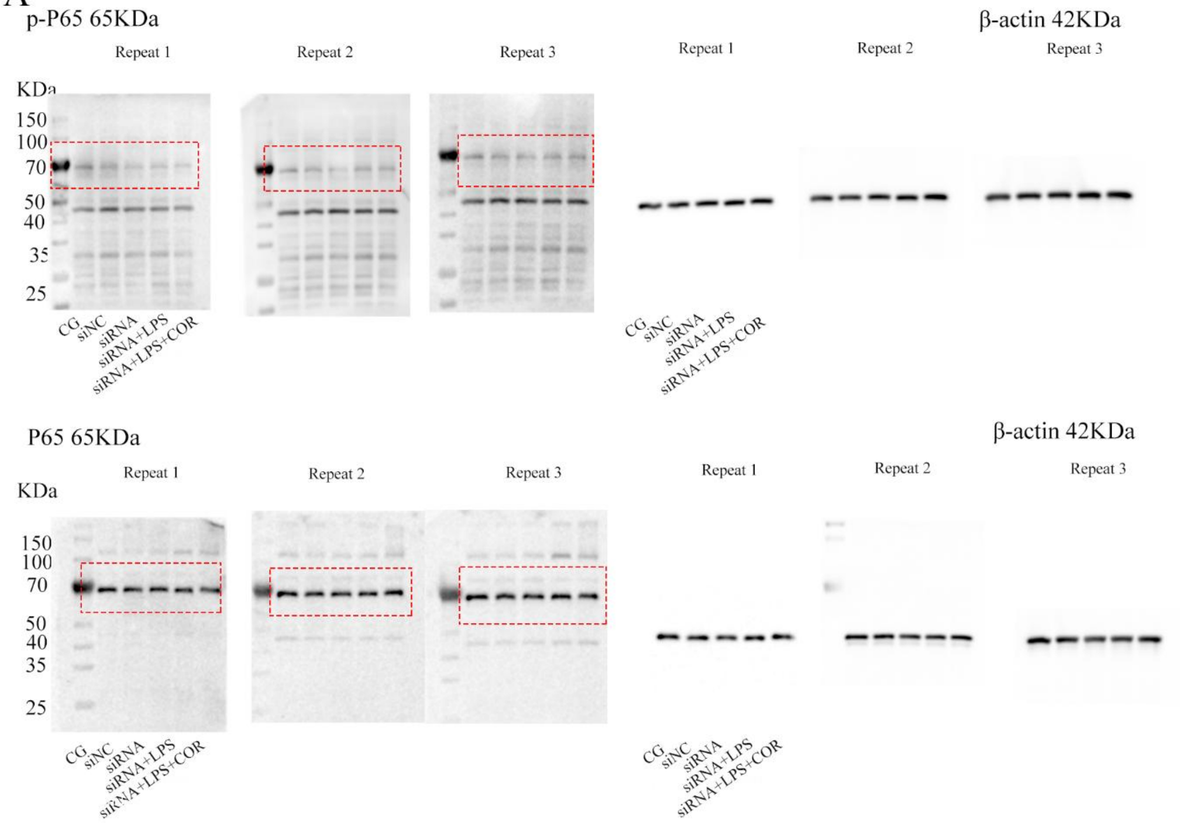

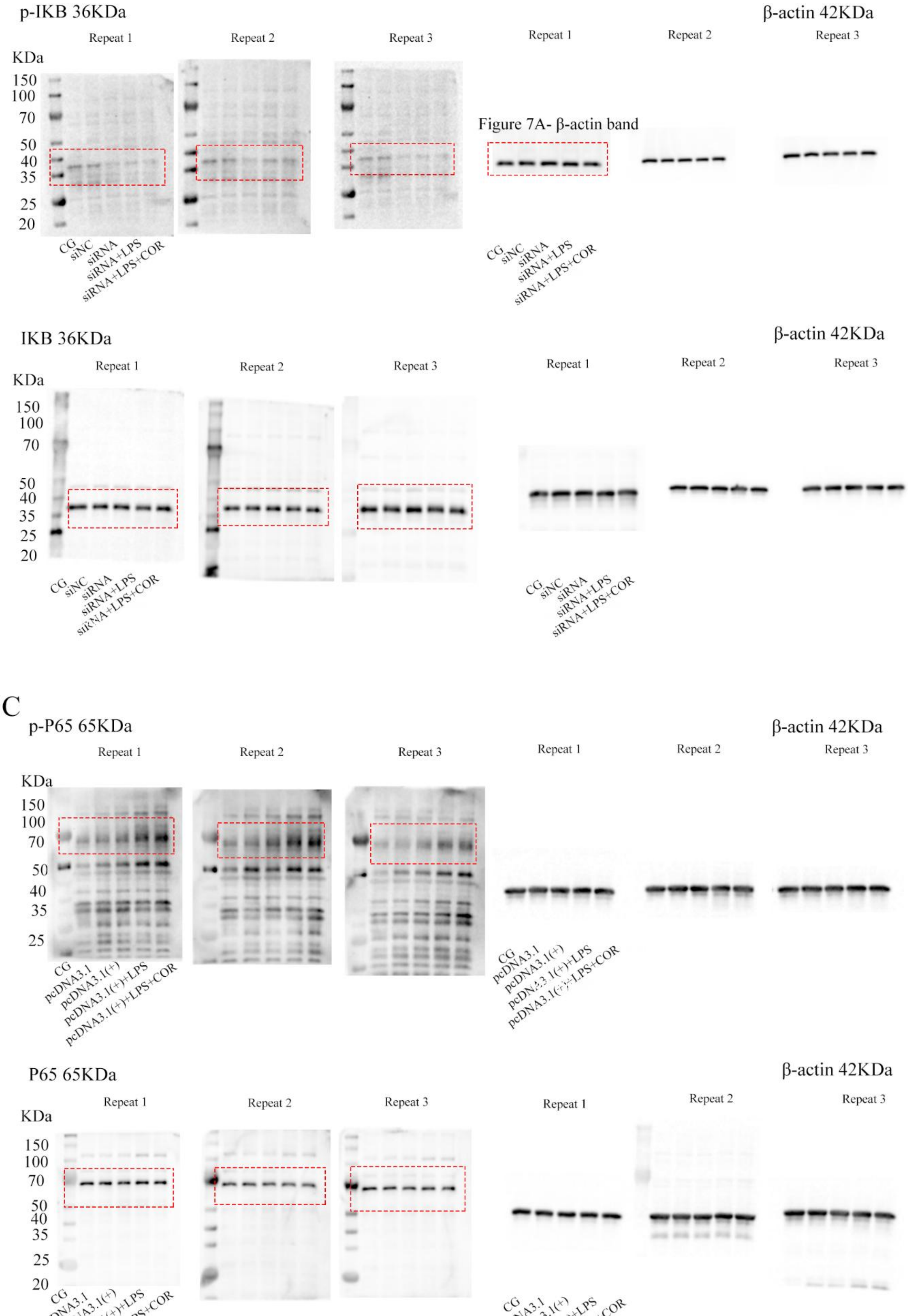
p-IKB 36KDa
β-actin 42KDa
Repeat 1
Repeat 2
Repeat 3
KDa
150
100
70
50
40
35
25
20
Figure 7A- β-actin band
CG
siNC
siRNA
siRNA+LPS
siRNA+LPS+COR
IKB 36KDa
β-actin 42KDa
C
p-P65 65KDa
β-actin 42KDa
pcDNA3.1
pcDNA3.1(+)
pcDNA3.1(+)+LPS
pcDNA3.1(+)+LPS+COR
P65 65KDa
β-actin 42KDa

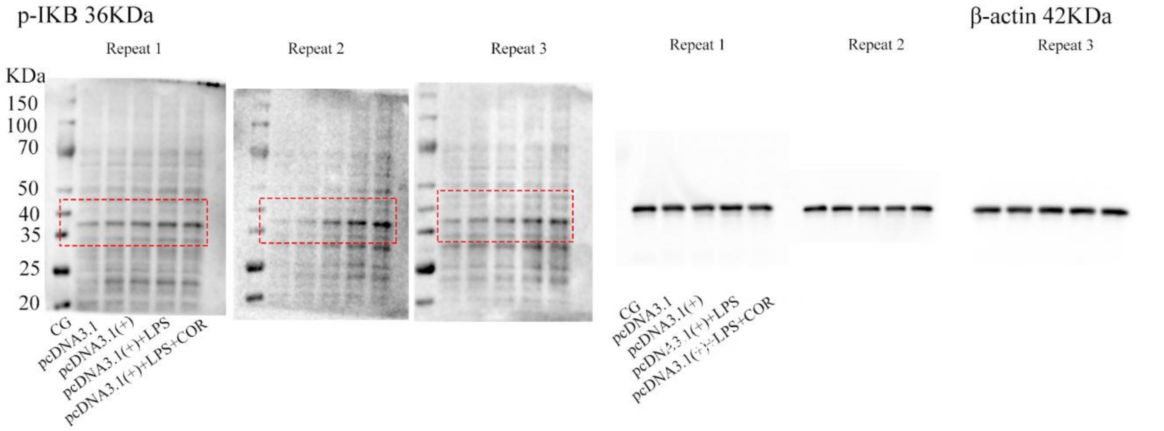

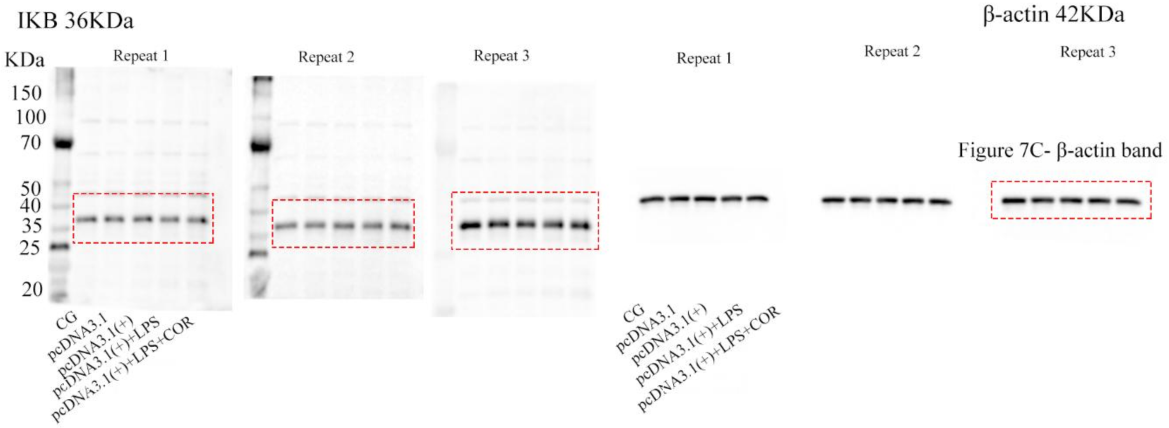

Figure 8 Original western blot for three repeats

A

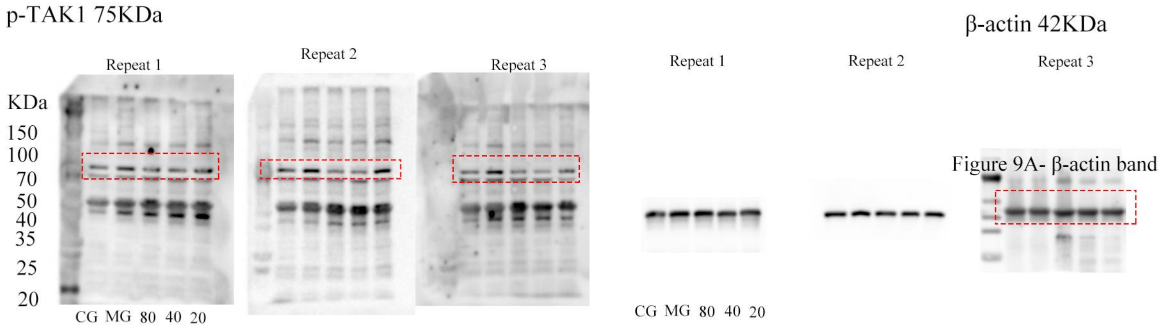

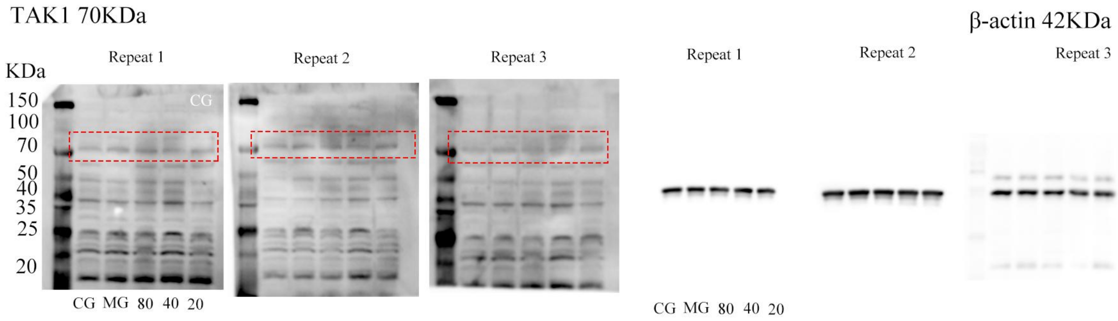

B

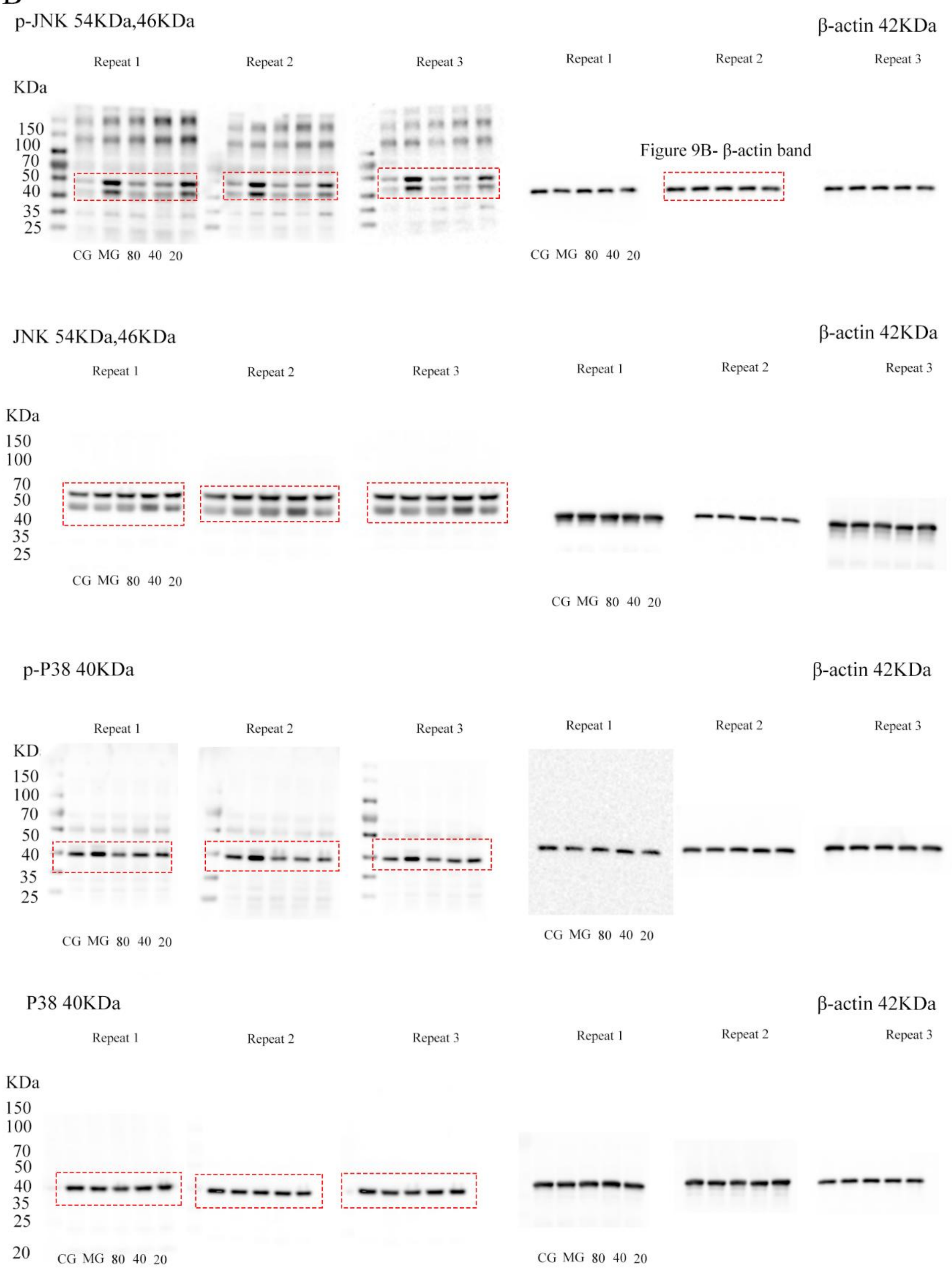
p-JNK 54KDa,46KDa
β-actin 42KDa
Repeat 1
Repeat 2
Repeat 3
KDa
150
100
70
50
40
35
25
CG MG 80 40 20
Figure 9B- β-actin band
JNK 54KDa,46KDa
p-P38 40KDa
P38 40KDa
20

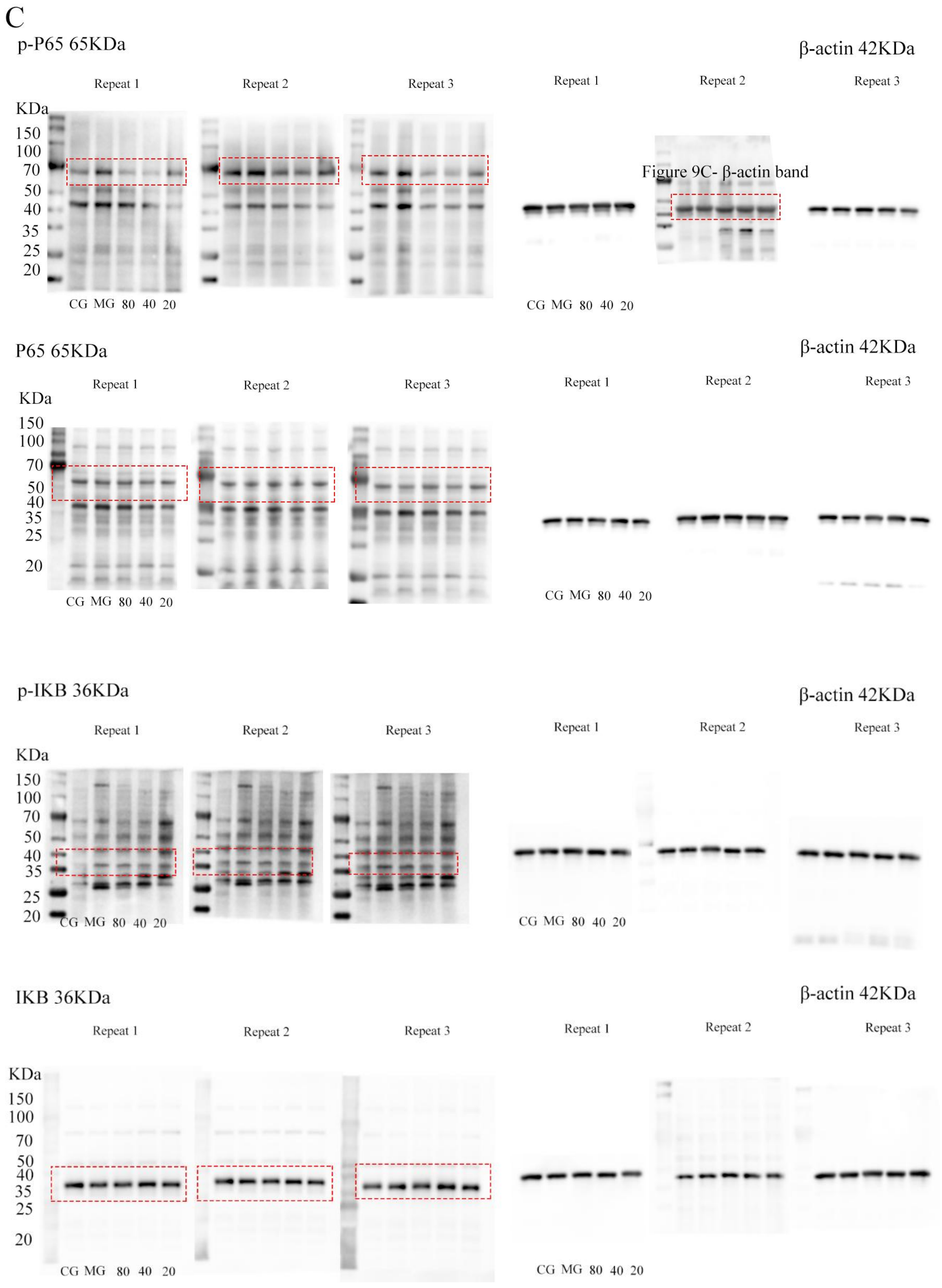
C
p-P65 65KDa
β-actin 42KDa
Repeat 1
Repeat 2
Repeat 3
KDa
150
100
70
50
40
35
25
20
CG MG 80 40 20
Figure 9C- β-actin band
P65 65KDa
β-actin 42KDa
p-IKB 36KDa
β-actin 42KDa
IKB 36KDa
β-actin 42KDa
